# Intervention Programs Targeting the Mental Health, Professional Burnout, and/or Wellbeing of School Teachers: Systematic Review and Meta-Analyses

**DOI:** 10.1007/s10648-023-09720-w

**Published:** 2023-03-01

**Authors:** Joanne R. Beames, Samantha Spanos, Anna Roberts, Lauren McGillivray, Sophie Li, Jill M. Newby, Bridianne O’Dea, Aliza Werner-Seidler

**Affiliations:** 1 Black Dog Institute, University of New South Wales, Sydney, NSW Australia; 2 Black Dog Institute and School of Psychology, University of New South Wales, Sydney, NSW Australia

**Keywords:** Teacher, Mental health, Burnout, Wellbeing, Review, Meta-analysis

## Abstract

This paper outlines a systematic review and meta-analyses to identify, describe, and evaluate randomised and non-randomised controlled trials of psychological programs targeting the mental health, professional burnout, and/or wellbeing of school classroom teachers. Eighty-eight unique studies were identified for inclusion in the review, and of those 46 were included in the meta-analyses (23 randomised controlled trials). In randomised controlled trials, the programs examined had large effects on stress (*g*=0.93), and moderate effects on anxiety (*g*=0.65), depression (*g*=0.51), professional burnout (*g=*0.57), and wellbeing (*g*=0.56) at post. In non-randomised controlled trials, programs had moderate effects on stress (*g=*0.50), and small effects on anxiety (*g*=0.38) and wellbeing (*g*=0.38) at post. Studies were heterogeneous in design and methodological quality was generally poor, particularly in non-randomised controlled trials. There was an inadequate number of comparisons to perform sub-group analyses, meta-regression, or publication bias analyses. Most of the programs examined required significant time, effort, and resources to deliver and complete. These programs may not translate well outside of research trials to real-world contexts due to teachers being time-poor. Priorities for research include using methodologically rigorous designs, developing programs for teachers with teachers (i.e. co-design), and considering implementation factors to ensure feasibility, acceptability, and uptake. Systematic Review Registration Number: PROPSERO - CRD42020159805.

**Supplementary Information:**

The online version contains supplementary material available at 10.1007/s10648-023-09720-w.

School teachers are a vulnerable workforce. International research shows that teachers consistently report burnout, psychological distress, anxiety, depression, fatigue, reduced self-confidence, and damaged personal relationships (García-Carmona et al., 2019; Thomson & Hillman, 2020). For example, a large proportion of secondary school teachers experience moderate-to-high levels of burnout, with 28.1% suffering from severe emotional exhaustion (García-Carmona et al., 2019). Poor mental health, burnout, and low wellbeing, have negative consequences for teacher performance and workforce retention, as well as for student learning and achievement (Sorensen & Ladd, 2020; Travers, 2017). Teachers who report high work-related stress are more likely to report intentions to leave the profession within five years (Thomson & Hillman, 2020), are less satisfied with and committed to their profession, and are less able to support their students emotionally and academically (Travers, 2017). Given the prevalence and negative impact of these factors, finding ways to improve the mental health, burnout, and wellbeing of teachers is crucial to sustain and protect the workforce.

Mental health can be conceptualised as more than just the absence of symptoms or clinical disorders (e.g. depression, anxiety; World Health Organisation, 2004). Prominent psychological theories position wellbeing and positive indicators of mental health as core components of what it means to be mentally healthy. For example, the Dual Factor Model proposes that complete mental health includes both the absence of negative factors (e.g. depression, anxiety, burnout) and the presence of positive factors (e.g. quality of life, work and life satisfaction, overall wellbeing; Greenspoon & Saklofske, 2001; Trompetter et al., 2017). A similar idea is presented in the Complete State Model of Mental Health, whereby psychological health is characterised both by the absence of mental illness and the presence of flourishing (Keyes & Lopez, 2002). Extant research on school teachers has typically focused on either mental illness or wellbeing in isolation, overlooking the well-established idea that all facets are important for mental health and functioning. To address this gap, the current paper describes and quantifies the effects of programs for school classroom teachers across the dimensions of both mental health and wellbeing.

There are a variety of programs that have been implemented to address teacher mental health, professional burnout, and/or wellbeing. Program types that have typically been delivered to teachers have included psychological approaches, such as psychoeducation, mindfulness, cognitive-behavioural skills, acceptance-based skills, behavioural stress management skills (e.g. relaxation), and other socio-emotional/interpersonal skills. Two systematic reviews of randomised controlled trials (RCTs) and non-randomised controlled trials (nRCTs) examined a range of programs and psychological outcomes for school teachers at various education levels (e.g. elementary, middle, and secondary schools). The first review (*k*=24) found that mindfulness, behavioural, and cognitive-behavioural programs were effective in reducing stress and burnout (von der Embse et al., 2019). The second review (*k=*29) found that wellbeing programs were generally effective for teachers, especially positive psychology interventions and brief activities to provoke changes in thinking and feeling (Dreer & Gouasé, 2021). Other systematic reviews and meta-analyses have also provided promising results for mindfulness-based programs (Emerson et al., 2017; Hwang et al., 2017; Klingbeil & Renshaw, 2018; Lomas et al., 2017; Zarate et al., 2019) and burnout (e.g. burnout; Iancu et al., 2018; Oliveira et al., 2021). In addition to the different theoretical approaches and content (e.g. types of learned skills) of available programs, there is marked variety in program duration, session length and frequency (Dreer & Gouasé, 2021; Iancu et al., 2018; Oliveira et al., 2021; von der Embse et al., 2019; Zarate et al., 2019), and delivery settings (Klingbeil & Renshaw, 2018). Although there is some evidence for the effectiveness of available programs, program heterogeneity means that it is unclear what components are driving the reported effects.

Another converging finding across published reviews is that there is heterogeneity in study methodology and level of rigour. Common sources of variation include sample size, target population (e.g. a combination of different types of teachers and other educators/school staff, different school levels), length of follow-up, operationalisation of outcome variables, and the assessment measures used. Many reviews have descriptively commented on lack of methodological rigour, with a particular issue being incomplete reporting of data or incomplete reporting in general (e.g. Oliveira et al., 2021). Five reviews have used a formal tool to assess risk of bias in included studies; four concluded that the overall quality of studies assessing mindfulness interventions were varied (Emerson et al., 2017; Hwang et al., 2017; Lomas et al., 2017; Zarate et al., 2019); the other concluded that the overall quality of studies assessing burnout interventions was acceptable, with greater than two thirds of studies at low risk of bias for at least three or more criteria (Iancu et al., 2018). While this provides some indication of the quality of studies assessing mindfulness and burnout interventions, a formal evaluation of a more comprehensive set of psychological programs for mental health, wellbeing, and/or professional burnout has not yet been conducted. Two additional gaps in the literature remain unaddressed that relate to program type and specificity of the teaching populations under investigation. Past reviews have included programs designed to build classroom competencies or student wellbeing (e.g. behavioural classroom management, teacher consultation dyads, vocal health; Dreer & Gouasé, 2021; Iancu et al., 2018; von der Embse et al., 2019). The primary target of these programs is the student body, with the rationale that the teachers who implement them may experience secondary mental health and wellbeing benefits. Programs for building classroom competencies are conceptually different to psychological programs that directly target teachers’ thoughts, feelings, and responses to stressful situations. They are not designed to address unique work-related challenges or psychological experiences that teachers experience and are structured and delivered in a different way. An in-depth examination of psychological programs for teachers is needed to describe what they involve and to quantify how effectively they improve mental health, professional burnout, and/or wellbeing for the teaching workforce. There is also a lack of specificity about the teaching population under investigation. Past reviews generally used a broad definition of educators and school personnel, including teachers, counsellors/psychologists, principals, paraprofessionals, administration staff, pastoral carers, and/or parents, and did not differentiate program effects between these groups (e.g. Iancu et al., 2018; Klingbeil & Renshaw, 2018; von der Embse et al., 2019). Focusing specifically on classroom teachers (i.e. those that have a primary responsibility for teaching) is important because they face role-specific challenges that influence appropriateness, uptake, and effectiveness of the programs designed specifically for their use.

## Aims

The aim of the current review was to identify, describe, and evaluate controlled trials (both randomised and non-randomised) of psychological programs targeting the mental health, professional burnout, and/or wellbeing of school classroom teachers. The aim of the meta-analyses were to quantify the effect sizes of programs to evaluate effectiveness and explore potential moderators of those effects.

Our approach extends the existing reviews in the field by evaluating a comprehensive range of programs and outcomes, as well as study designs, and focusing on programs for the teaching workforce. We explore a more detailed set of program and study characteristics to further identify sources of heterogeneity. One critical area of differentiation is our focus on program features related to feasibility, including delivery method and setting, and the type of support that teachers or schools receive to complete the programs. Furthermore, including both RCTs and nRCTs is a strength of our approach. Given that a large proportion of studies in this field are non-randomised, their inclusion offers a wealth of information about available programs and their effectiveness in real-world contexts. We evaluated methodological rigour of RCTs and nRCTs by using established tools to evaluate study quality and risk of bias of the included studies.

## Methods

### Protocol and Registration

Consistent with Preferred Reporting Items for Systematic Reviews and Meta-Analyses (PRISMA) guidelines (Moher et al., 2009), this review was prospectively registered with PROSPERO on 27th November 2019 (CRD42020159805).

### Systematic Review Eligibility Criteria

#### Types of Participants

Participants were teachers, from pre-service (i.e. teaching candidates pursuing formal qualifications/licences) to experienced levels, who had a primary responsibility for educating students (i.e. classroom teachers, special education teachers). Examples include early childhood teachers (e.g. pre-school), elementary/primary school teachers (Kindergarten/Preparatory to Grade 6), middle school teachers (e.g. Grades 6 to 8), and secondary/high school teachers (i.e. Grades 7 to 12). The terms early childhood, elementary, middle, and secondary school will be used throughout this review to ensure consistency in terminology. Tertiary education or vocational teachers were not included. Studies that combined an eligible teacher sample with another sample (e.g. students, parents) were included in the review if teachers comprised >50% of the sample.

#### Types of Programs

Eligible programs included any psychological program delivered to the identified population that aimed to impact their mental health, professional burnout, and/or wellbeing outcomes. These programs were defined as targeting thoughts, feelings, and/or responses to stressful situations. Examples include cognitive-behavioural therapy, mindfulness-based stress reduction, and emotional-skills training. Programs that were not delivered to teachers or did not directly aim to impact their mental health, burnout, and/or wellbeing outcomes were excluded. To be included in the review, the psychological component/s of multi-component programs had to comprise >75% of the program.

#### Types of Control Group

Studies were included in the review if they included any type of control (e.g. no-intervention or treatment as usual, waitlist control, attention control) or comparison group.

#### Types of Outcomes

Studies were included if they reported outcomes for teachers that had a clear mental health, professional burnout, and/or wellbeing component. Mental health symptoms or diagnoses included anxiety, depression, stress, psychological distress, post-traumatic stress-disorder, secondary traumatic stress or vicarious trauma, somatisation, and sleep problems. Wellbeing outcomes included subjective wellbeing and quality of life, where subjective wellbeing refers to cognitive and/or additive appraisal of one’s life (e.g. life satisfaction, work satisfaction). Secondary outcomes, including resilience, positive and negative affect, and fatigue, were included from studies that met all other inclusion criteria. All outcome measures needed to be valid and use reliable rating scales suitable for adults or teaching professionals.

#### Types of Studies

Quantitative studies were included if they included any type of control or comparison group, namely RCTs, including cluster RCTs and nRCTs. Studies were included if they were published in the English language in peer-reviewed journals. Studies without a control or comparison group, studies that were purely descriptive or qualitative, and protocol papers were excluded.

### Search Strategy

Consistent with the PRISMA statement, we conducted a systematic search of three electronic databases: PsychINFO, MEDLINE, and EMBASE. We used a combination of keywords and Medical Subject Heading (MeSH) terms related to (a) teachers, (b) mental health, professional burnout, and/or wellbeing outcomes, and (c) interventions/programs. The search was conducted on 11th of February 2020. See Appendix A in the Supplementary Material for search strategy used for PsychINFO. The search was adapted to meet the requirements of each database. No limits were applied to the search. This search was re-run on the 13th November 2020 and again on the 30th November 2021 to identify recently published articles.

### Data Extraction and Management

Study characteristics and outcomes were extracted by JRB, SS, and AR and entered into a Microsoft Excel spreadsheet. A smaller subset of extracted data (10%) was checked independently by AW-S. There were no discrepancies. We extracted the following data when reported: author, year of publication, country of study, allocation level, control/comparison group, reimbursement, teacher type, universal (delivered to all teachers regardless of initial symptom levels) or indicated (delivered to teachers with elevated mental health symptom levels) sample, sample size, percent attrition from the program, mean age, percent female, program name and details (i.e. content, delivery method and setting, length, number of sessions, format, facilitator, home tasks, program specificity to teachers), and outcomes. Program content was categorised based on the primary theoretical approach (e.g. mindfulness and/or relaxation, cognitive-behavioural skills, socio-emotional, relationship and/or interpersonal skills). Program specificity to teachers refers to whether the program was developed/adapted specifically for teachers or the school context or whether it was developed for another group/purpose and applied to teachers without adaptation. We also extracted length of follow-up for both the program and control groups when available. We extracted data for post-intervention (<1 month), short-term (1–6 months inclusive), and/or long-term (>6 months) follow-up. These categories were based on the time-periods most frequently reported by the authors. Few studies reported intervention adherence or fidelity, school type and location, and so they are not considered further.

To descriptively compare the effectiveness of programs identified in the review, standardised effect size estimates were calculated using Hedges’s *g* when raw data were available (see “Calculation of Effect Sizes” in the Meta-Analytic Procedures section). When raw data were not available, Cohen’s *d* effect size estimates were extracted when they were reported in the primary papers. These effect sizes compare the program versus the control/comparison at post and follow-up for each outcome variable. Positive effect estimates indicate that the intervention group improved more, or had better outcomes, than the control/comparison group. We report Hedges’s *g* for select study and program characteristics at post throughout the results below.

### Quality and Risk of Bias

We used the Cochrane Collaboration Risk of Bias tool for RCTs (ROB-II, Sterne et al., 2019) and for non-randomised intervention studies (ROBINS-I, Sterne et al., 2016) to assess the quality and risk of bias of studies included in the review. We selected domains based on relevance to psychology intervention trials.

#### ROB-II

We assessed bias in five domains: (1) randomisation (e.g. whether allocation and concealment procedures of participants produced comparable groups), (2) deviations of intended intervention assignment (e.g. whether participants and research personnel were blinded to group allocation, whether there are systematic differences between the care provided to groups beyond the assigned interventions), (3) missing outcome data (e.g. whether missing data reflected systematic differences between groups and produced biased effect estimates), (4) measurement of the outcome (e.g. whether participant responses were biased due to knowledge of group allocation), and (5) selective reporting of data (e.g. whether all outcomes were appropriately reported). All papers were independently coded by two authors (JRB, SS, LM, BO, SL, and/or AR), and the team resolved disagreements through discussion.

#### ROBINS-I

We assessed bias in six domains: (1) bias due to confounding (e.g. whether pre-existing factors that predict whether an individual receives one intervention or another are measured without error and controlled for in study design or statistical techniques), (2) bias in selection of participants into the study (e.g. whether selection of participants is related to both the intervention and the outcome), (3) bias due to deviations from intended interventions (e.g. whether there are systematic differences between the care provided to groups beyond the assigned interventions), (4) bias due to missing data (e.g. whether the proportion and reasons for missing data are similar for all groups), (5) bias in measurement of outcomes (e.g. whether participant responses were biased due to knowledge of group allocation, whether methods were comparable across groups), and (6) bias in selection of the reported result (e.g. whether all outcomes were appropriately reported). Ratings were made independently by JRB and LM, and the authors resolved disagreements through discussion and consultation with AW-S.

## Meta-Analytic Procedures and Analyses

### Inclusion Criteria

The meta-analyses involved more stringent criteria to enable stronger quantitative conclusions about effectiveness of programs for teachers. For studies that combined an eligible teacher sample with another ineligible sample, they were only included in the meta-analyses if separate data was available for the eligible teaching subsample. Studies that used a comparison rather than a control group were included in the review for completeness, but not the meta-analyses.

### Data Extraction

For studies meeting inclusion criteria, we extracted means, standard deviations, and sample size of completers at post-intervention, and short- and long-term follow-up. When means and standard deviations were not available, we extracted mean difference scores and independent groups’ *p*-values. In studies in which appropriate outcome data were not reported, JRB contacted the authors to obtain this information.

For studies that included data from total scale scores and component subscales, we only included relevant subscales (e.g. the Anxiety, Depression, and Somatisation Subscales of the Brief Stress Inventory were included, but not the Global Symptom Severity Index). The decision to preferentially include relevant subscales aligns with our overall aim of exploring program effects on specific mental health symptoms, professional burnout, and/or wellbeing. For studies that included two measures of the same outcome domain (e.g. the Perceived Stress Scale and the Depression, Anxiety and Stress Scale — Stress Subscale), we included the most used measure across studies to facilitate consistency and to increase the sample size for the component meta-analyses. Note that we did not include two (or more) measures from the same study in an outcome effect size estimate to avoid the introduction of systematic bias from over-representing some studies over others. For the Maslach Burnout Inventory, we used the emotional exhaustion subscale rather than the cynicism or depersonalisation subscales. Emotional exhaustion is the key psychological dimension of burnout that is characterised by a stress reaction related to work demands and overload (Maslach & Leiter, 2016). Exhaustion is conceptually similar to a conventional stress variable and is more predictive of stress-related health outcomes compared to the other two dimensions (Maslach & Leiter, 2016). For studies that included multiple wellbeing measures, we selected broader measures of wellbeing to capture greater dimensionality rather than specific measures of singular constructs (e.g. satisfaction). Finally, because the focus of our review was on a workplace setting, we selected work satisfaction measures over life satisfaction measures when both were reported in a single study.

### Calculation of Effect Sizes

We used Comprehensive Meta-Analysis (version 3.0, Biostat Inc.) to calculate individual study and pooled effect sizes. For each comparison between an intervention and control group, we calculated the standardised mean difference at post-intervention adjusted for small samples (Hedges’ g; Hedges & Olkin, 2014). This calculation involves subtracting the average score of the intervention group at post-intervention from the average score for the control group at post-intervention and dividing the result by the pooled standard deviation of the two groups (i.e. controlled effects). Effect sizes of 0.2, 0.5, and 0.8 refer to small, moderate, and large effect sizes, respectively (Cohen, 2013). We also calculated the 95% confidence interval around effect sizes. For studies that had multiple control groups, we divided the number of participants in the intervention group by the number of control groups such that each participant was only represented once in the meta-analyses. We used a random effects model because considerable variation in estimated effect sizes between studies was expected (i.e. heterogeneity). There are many potential sources of heterogeneity, including differences in interventions, the population, the study design, or the data analysis method. The random effects model assumes that the true effect size varies from one study to the next, and therefore permits the generalisation of the corrected effect size estimate to the broader population (Borenstein et al., 2009).

We conducted meta-analyses separately for RCTs and nRCTs. For primary outcomes across both types of trials, we calculated controlled post-intervention effect sizes for (i) anxiety, (ii) depression, (iii) stress, (iv) professional burnout, and (v) wellbeing. We also calculated controlled post-intervention effect sizes for psychological distress in RCTs and for somatisation in nRCTs. For secondary outcomes, we calculated controlled post-intervention effect sizes for positive and negative affect across both types of trials, and effect sizes for resilience in nRCTs. Short-term follow-up controlled effects were calculated for outcomes with enough studies (i.e. *k*>1) to facilitate comparison: for RCTs, outcomes included anxiety, stress, sleep, professional burnout, wellbeing; for nRCTs, outcomes included anxiety, depression, stress, professional burnout, and wellbeing. There were not enough studies (i.e. *k=*1) to calculate effect sizes for the other primary and secondary outcome variables at post-intervention, short-term, or long-term follow-up. The significance level for these analyses was set at *p*<0.008 following a Bonferroni correction (*k=*6, the number of primary outcomes evaluated for each type of trial).

### Heterogeneity

We calculated the *I*^2^ statistic to test the heterogeneity of effect sizes at post-intervention. *I*^2 ^estimates the proportion of variance in study estimates that is due to heterogeneity and is independent of the number of studies included in a meta-analysis (Higgins & Thompson, 2002). Given these characteristics, *I*^2 ^arguably provides a more informative measure of heterogeneity than other approaches (e.g. Cochrane’s Q; Higgins & Thompson, 2002). *I*^2 ^values are commonly expressed as percentages, whereby 25%, 50%, and 75% correspond to low, medium, and high levels of heterogeneity, respectively (Higgins & Thompson, 2002). The 95% confidence interval limits were also calculated to estimate dispersion in the observed effect sizes.

### Additional Analyses

Additional analyses were planned; however, there was an inadequate number of comparisons to perform sub-group analyses, meta-regression, or publication bias analyses for each of the primary outcomes. Guidelines suggest that at least 10 studies are needed in a meta-analysis to adequately power tests of moderation and funnel plot asymmetry (Higgins, updated February 2022; Tanner-Smith & Grant, 2018).

## Descriptive Results

### Selection of Studies

See Appendix B in the Supplementary Material for the PRISMA diagram illustrating the study selection process. A total of 19,600 articles were identified, from which 3816 duplicates were removed. In the first step, both titles and abstracts of 15,784 articles were screened for eligibility using the inclusion/exclusion criteria. JRB screened articles from the original search. JB, SS, and AR screened articles from the updated searches. In total, 15,573 articles did not meet the inclusion criteria and therefore were excluded. In the second step, JRB, SS, and AR independently screened the full text articles of the remaining 211 records. Of these records, 122 were excluded because they did not meet the inclusion criteria. Any disagreements were resolved through discussion and consultation with AW-S. Screening resulted in 89 articles (49 RCTs, 40 nRCTs) being included in the current review. One article (Jennings et al., 2019) reported follow-up data of an RCT reported separately (Jennings et al., 2017), leaving 88 original studies included in the review. Of these articles, 46 were included in the meta-analyses (23 RCTs, 23 nRCTs).

### Study Characteristics

See Tables S1–S2 and S3–S4 in Appendix C of the Supplementary Material for characteristics and effect sizes of included RCTs and nRCTs, respectively. The 88 unique studies included in the current review had a total of 8763 participants. Sample sizes were generally small, with a median of 63 participants (range: 14 to 961). For primary mental health outcomes, 31 studies examined anxiety (35.23%, *g*=0.36^1^), 23 examined depression (26.14%, *g*=0.31), 48 examined stress (54.55%, *g*=0.59), six examined psychological distress (6.82%, *g*=2.41), one examined trauma (1.14%, *g*=0.22), three examined somatisation (3.41%, *g*=0.61), eight examined sleep (9.09%, *g*=0.71), 40 examined professional burnout (45.45%, *g*=0.55), and 24 examined wellbeing including work/life satisfaction (27.27%, *g*=0.5). For secondary outcomes, five studies examined resilience (5.68%, *g*=0.29), 14 examined positive/negative affect (15.91%, *g*=0.21 and *g*=0.37, respectively), and three examined fatigue (3.41%, *g*=0.4). Across the entire set of studies, effect sizes ranged from *g*=−0.87 to *g*=9.44 and were descriptively larger for RCTs (*g=*0.63) compared to nRCTs (*g=*0.33).

### Control Group

Most studies included a control group (*n*=75, 85.23%, *g*=0.65), with 11 evaluating the intervention against a comparison group such as another individual psychological intervention or a classroom-management style intervention (12.50%, *g*=0.25). Two studies did not clarify whether they used a control or comparison group (2.27%). Most studies used a passive control group (*n=*67, 89.33%, *g=*0.66), including a waitlist control (*n=*37, 55.22%, *g*=0.7) or a no-intervention control (*n=*31, 46.27%, *g*=0.63). No-intervention control groups were those that did not receive the formalised intervention at any point during the study. Three studies used an active control (4%), and one study used a combination of passive and active control groups (1.33%). Three studies did not specify the type of control group (4%).

### Population Characteristics

Most studies were conducted in the USA or Canada (*n=*29, 32.95%), followed by Europe (*n=*21, 23.86%) and Asia (*n=*20, 22.73%). Most studies exclusively recruited school teachers that had a primary teaching role (*n*=65, 73.86%, *g=*0.62), although 21 studies included other school professionals (e.g. counsellors, principals, administration staff, assistants; 23.86%, *g=*0.51) and two studies did not specify the type of teaching sample (2.27%). There were a variety of teaching levels investigated. Just under one third of the studies included a combination of teaching levels (e.g. pre-school, elementary, and secondary (*n=*25, 28.41%, *g*=0.35), while 19 focused on secondary levels (21.59%, *g*=0.66), 16 focused on elementary levels (18.18%, *g=*0.48), and two focused on middle school levels (2.27%). Fewer studies focused on pre-service (*n=*10, 11.36%), special education (*n=*6, 6.82%), and early childhood (*n=*2, 2.27%), and eight studies did not provide any information about teaching level (9.09%). Most studies delivered programs universally (*n=*77, 87.50%, *g*=0.45); 11 studies delivered programs to indicated samples (12.50%, *g*=1.36). The samples were predominantly female (79.08%, range=32.26–100%), with a mean age of 39.70 years. See Tables S1 and S3 for additional details about the population.

### Randomisation

The largest proportion of studies randomised teachers at the individual level (*n*=65, 73.86%, *g*=0.68), followed by randomisation at the school level (*n*=16 studies, 18.18%, *g*=0.31). Four studies randomised at the classroom level (4.55%), two randomised at the university/college level (i.e. for pre-service school teachers; 2.27%), and one study did not report level of randomisation (1.14%).

### Program Characteristics

#### Program Content

Most studies examined a mindfulness and or/relaxation-based program (*n*=35, 39.77%, *g*=0.5), followed by cognitive-behavioural skills (*n*=14, 15.91%, *g*=0.61), stress management skills (*n*=13, 14.77%, *g=*0.38), and then socio-emotional, relationship and/or interpersonal skills (*n=*8, 9.09%, *g*=0.48). Fewer studies included rational emotive behaviour therapy programs (*n*=5, 5.68%) or acceptance and commitment therapy programs (*n=*2, 2.27%). A group of studies used strategies that did not fall into these categories (*n=*12, 13.64%, *g*=0.43). This group of studies combined many different theoretical approaches to psychological therapy (e.g. acceptance and commitment, cognitive-behavioural, visualisation, and relaxation skills), or focused on gratitude skills, work environment strategies, sleep education, or transpersonal psychology. Most programs were context-specific, meaning that they were developed/adapted for teachers (*n=*67, 76.14%, *g*=0.63). These programs typically included strategies that addressed stressors and experiences characteristic of the teaching role and workplace environment, as well as strategies that addressed teacher mental health issues. Other programs were context-general, applied to teachers without any adaptation to their role or workplace (*n=*18, 20.45%, *g*=0.48). Three studies did not provide enough information to categorise them (3.41%). Most studies reported on home task requirements of the programs (*n=*75, 85.23%). Home tasks were varied in terms of length and duration, ranging from 30 to 40 min of formal mindfulness practice each day for 5-weeks (Vesely et al., 2014) to conducting progressive muscle relaxation each day for 8 min throughout the program (Shimazu et al., 2003).

#### Format and Delivery

Most studies included programs that were conducted face-to-face (*n=*79, 90.80%, *g*=0.57), rather than online (*n=*5, 6.98%, *g*=0.49) or in a blended delivery form (*n=*2, 2.30%, *g*=1.19). Most programs were delivered exclusively in group format (*n*=69, 80.23%, *g*=0.58), rather than delivered to individuals (*n=*6, 6.98%, *g=*0.43) or using a hybrid format (i.e. group and individual components; *n=*11, 12.79%, *g*=0.38). Most studies included programs that were either school-based (*n=*28, 31.82%, *g*=0.61) or school-supported (*n=*19, 21.59%, *g*=0.58). School-based refers to a program that is endorsed by the school and delivered to teachers during school hours, or before/after school, on school premises. School-supported refers to a program that is endorsed by the school, but does not require additional school resourcing (e.g. time/funding). Most studies used programs that were delivered over 1–12 weeks (*n*=60, 68.18%, *g*=0.61); nine were delivered over less than 1 week (10.23%, g=0.29) and 14 were delivered over more than 12 weeks (15.91%, *g=*0.69). Furthermore, most programs involved 1–8 sessions (*n=*56, 63.64%, *g*=0.42); 18 involved 9–12 sessions (20.45%, *g*=0.76) and 9 involved more than 12 sessions (*n*=9, 10.23, *g*=0.65). Sessions typically lasted between 1 and 4 h (*n=*42, 59.09%, *g*=0.69). Other studies used brief sessions lasing less than an hour (*n=*11, 12.50%, *g*=0.68) or longer sessions lasting between 5 and 8 h (*n=*12, 13.64%, *g*=0.25).

Over half of studies identified the intervention facilitator (*n*=66, 75%). Of these studies, 59 used a facilitator that was external to schools (89.39%, *g*=0.57), including accredited or certified trainers (e.g. qualified mindfulness practitioner teachers), clinical psychologists, and researchers. Only three studies reported using a facilitator that was internal to the school context (4.55%, *g*=0.84), such as a teacher or nurse. Four studies did not require a facilitator because the programs were self-guided or delivered online (6.06%, *g*=0.42). See Tables S1 and S3 in Appendix C of the Supplementary Material for additional details about program format and delivery.

### Reimbursement

Most studies reported whether teachers received reimbursement for their participation (*n=*70, 79.55%), of which 48 did not provide any reimbursement (54.55%, *g*=0.58). For the studies that did report providing reimbursement (*n*=19, 21.59%, *g*=0.40), there was considerable variation ranging from professional development points or course credits, reduced pricing for the program, money, and a chance to earn gift certificates.

### Outcomes

A wide range of measures was used to assess primary outcomes, with a total number of 68 measures across the studies included in the review (not including individual subscales). See Tables S2 and S4 in Appendix C of the Supplementary Material. Stress and professional burnout were the most consistently measured outcomes, with 24 studies using the Perceived Stress Scale (50%) and 31 studies using the Maslach Burnout Inventory (77.5%). The State Trait Anxiety Inventory — State Subscale was the most used measure for anxiety (*n=*11, 35.48%), and the Depression, Anxiety, Stress Scales — Depression Subscale was the most used measure for depression (*n=*8, 34.78%). There was no clear consistency in the measures used to assess psychological distress, somatisation, sleep problems, or wellbeing.

### Length of Follow-Up

For the first measurement point, 75 studies measured outcomes within 1-month of finishing the intervention (85.23%), 9 studies measured outcomes 1–6 months after post-test (10.23%), and two studies measured outcomes more than 6 months after post-test (2.27%). Only 30 studies reported relevant follow-up measures for both the control and program groups (34.09%). Of these studies, 27 were assessed 1–6 months after post-test (90%) and 2 were assessed more than 6 months after post-test (6.67%). One study included a follow-up measure but did not specify when the measurement took place (3.33%).

### Attrition

Over half of the studies reported percentage attrition from the program (*n*=65, 73.86%). Average attrition from the program group was 12.69%, ranging from 0 to 66.67%.

### Risk of Bias

#### ROB-II

Roughly half of the RCTs were assessed to have some concerns (*n=*26, 53.06%) and the other half were assessed to have high risk of bias (*n=*23, 49.94%). Most studies were assessed to have some concerns due to participants being aware of the assigned intervention (*n=*46, 93.88%) or due to an absence of a pre-specified analysis plan (*n=*47, 95.92%). High risk of bias was most typical in the missing outcome domain, with 28 studies not reporting enough information about dropout or not accounting for dropout (57.14%). Furthermore, 34 studies did not report enough information to ascertain whether intervention allocations could have been foreseen prior to or during enrolment (69.39%). Descriptively, studies that were assessed as having some risk of bias had a large effect size (*g*=1.04); studies that were assessed as having high risk had a medium effect size (*g*=0.59). See Table S5 in Appendix C of the Supplementary Material.

#### ROBINS-I

Most nRCTs were assessed to have serious risk of bias (*n=*35, 87.5%) and the remaining five were assessed to have critical risk of bias (12.5%). This result was primarily due to the outcome domain. All but one of the studies were assessed to have serious risk of bias because the measurement tools were self-report and the participants were likely aware of the intervention received (*n=*39, 97.5%). In addition, 18 studies had serious or critical risk of bias in the reporting domain because they lacked a clear analysis plan (45%), and 21 studies had serious or critical risk of bias in the confounding domain because they lacked experimental and statistical control of extraneous variables (52.5%). Nine studies did not report enough information about missing data to make a judgment about risk (22.5%). Descriptively, studies that were assessed as having serious and critical risk of bias had small effect sizes (*g*=0.41 and *g*=0.27, respectively). See Table S6 in Appendix C of the Supplementary Material.

## Meta-Analytic Results

Meta-analyses using random effects models were conducted for RCTs and nRCTs separately to compare the intervention and control groups on the outcomes at post-intervention and follow-up (i.e. controlled effects). Primary outcomes with enough RCTs and/or nRCTs to be meta-analysed at post included anxiety, depression, stress, psychological distress, somatisation, professional burnout, and wellbeing. Primary outcomes with enough RCTs and/or nRCTs to be meta-analysed at short-term follow-up included anxiety, depression, stress, sleep, professional burnout, and wellbeing. Secondary outcomes with enough studies to be meta-analysed at post included positive and negative affect (for RCTs and nRCTs) and resilience (for nRCTs). The significance threshold was set at *p*<0.008. All figures and tables are presented in Appendices B and C of the Supplementary Material.

### Randomised Controlled Trials

Controlled effects at post-intervention were significant and of a large size for stress (*g*=0.93), of a moderate size for anxiety (*g*=0.65), depression (*g*=0.51), professional burnout (*g*=0.57^2^), and wellbeing (*g*=0.56), and of a small-to-moderate size for positive affect (*g*=0.42). Post-intervention effects were non-significant at the conservative threshold for psychological distress. The short-term follow-up intervention effect sizes were significant and of a large size for stress (*g*=1.79), and of a moderate size for professional burnout (*g*=0.82); they were non-significant for anxiety, sleep, and wellbeing. See the Supplementary Material for all statistics (Table S7) and forest plots of post-intervention effects for primary outcomes (Fig. S2–S7).

#### Heterogeneity

Based on *1*^2^ statistics (see Table S8 in the Supplementary Material), there was high heterogeneity in the controlled effects at post-intervention for stress, psychological distress, and professional burnout. There was low heterogeneity for anxiety and depression, and moderate heterogeneity for wellbeing. The heterogeneity estimates for stress and professional burnout were estimated with some precision, whereas the other estimates were not. This imprecision likely reflects small sample sizes (von Hippel, 2015).

### Non-Randomised Controlled Trials

Controlled effects at post-intervention were significant and of a moderate size for stress (*g*=0.50), and of a small size for anxiety (*g*=0.38) and wellbeing (*g*=0.38). Effects were non-significant for depression, somatisation, professional burnout, positive and negative affect, and resilience. Controlled effects at short-term follow-up were non-significant for anxiety, depression, stress, professional burnout, and wellbeing. See the Supplementary Material for all statistics (Table S9) and forest plots of post-intervention effects for primary outcomes (Fig. S8–S13).

#### Heterogeneity

Based on *1*^2^ statistics (see Table S10 in the Supplementary Material), there was moderate to high heterogeneity in the controlled effects at post-intervention for anxiety, depression, stress, and wellbeing. The wide confidence intervals indicate that these estimates are imprecise. The heterogeneity estimates for professional burnout, somatisation, positive affect, negative affect, and resilience may not be reliable given the few comparisons contributing to the effect size (von Hippel, 2015).

## Discussion

We systematically reviewed the existing literature on psychological programs for the mental health, professional burnout, and/or wellbeing of school classroom teachers. We identified 88 unique studies with a total of 8763 participants for the review (including 48 RCTs and 40 nRCTs). Consistent with existing literature, there was wide variability in the programs in terms of theoretical approach, structure, and format. We also meta-analysed overall program effects on anxiety, depression, stress, psychological distress, somatisation, professional burnout, and/or wellbeing. We found that the psychological programs evaluated by these studies have positive effects for a range of mental health outcomes, as well as professional burnout and wellbeing, relative to control groups.

The meta-analyses showed that teacher programs are superior to control groups on most of the primary outcomes at the immediate post-test, with some effects sustained over the short-term (1–6 months). The size of the immediate effects varied. In RCTs, evaluated programs had significant large effects on stress (*g*=0.93), and moderate effects on anxiety (*g*=0.65), depression (*g*=0.51), professional burnout (*g=*0.57), and wellbeing (*g*=0.56) compared to controls. In nRCTs, programs had significant moderate effects on stress (*g=*0.50), and significant small effects on anxiety (*g=*0.38) and wellbeing (*g=*0.38) compared to controls. The effect size for stress and professional burnout increased in size for RCTs over the short-term compared to controls (*gs=*1.79 to *g*=0.82). However, few studies evaluated sustained effects over time. Follow-up measurements are necessary to evaluate whether effects persist or deteriorate over time. Overall, these findings are generally consistent with other meta-analyses that investigated the controlled effects of different programs on teacher mental health, professional burnout, and/or wellbeing outcomes (Iancu et al., 2018; Klingbeil & Renshaw, 2018; Oliveira et al., 2021; Zarate et al., 2019).

Despite the promising findings from our meta-analyses, there was considerable heterogeneity in the included studies. Confidence intervals either could not be estimated due to small samples or indicated that heterogeneity estimates were imprecise. Exceptions to this pattern were the stress and professional burnout outcomes in RCTs, for which heterogeneity was high and estimated with precision. In these cases, high heterogeneity most likely captures the cross-disciplinary and emerging nature of the field. The implication is that researchers are using different programs, research designs, methods, and analysis techniques to explore effects on teacher outcomes. Support for this explanation comes from our review findings. Of note, there was a lack of consistency in how mental health, professional burnout, and/or wellbeing outcomes were measured, and the types of programs being evaluated. Programs were eclectic in terms of duration and frequency of sessions (i.e. intensity), delivery setting and level of school involvement, learned strategies, and the theory underpinning the treatment model (e.g. cognitive-behavioural principles, mindfulness principles, demands-resources model of coping, transpersonal theory). This finding converges with other descriptive systematic reviews and meta-analyses investigating teacher mental health programs (e.g. Dreer & Gouasé, 2021; Klingbeil & Renshaw, 2018; von der Embse et al., 2019).

To supplement our meta-analysis and describe effect sizes across all studies included in our review, we computed averaged effect sizes for different study and program characteristics. There was wide variation in the effect size estimates across all studies (*g*=−0.87 to *g*=9.44). In terms of study characteristics, those that used control groups, classroom teachers from secondary levels, and indicated samples with some level of pre-existing symptomatology or lower wellbeing had larger effects. In terms of program characteristics, those that used cognitive-behavioural skills and were adapted/developed specifically for school teachers had larger effects than other programs. Programs that were delivered in group format, in schools, for more than 1 week also had comparably larger effect sizes. We also found positive effects for briefer sessions (e.g. lasting less than an hour) and fewer sessions (e.g. 1–8 sessions), although more improvement might be associated with a greater number of shorter sessions. These results indicate that setting a realistic amount of time aside for staff to address their mental health and wellbeing in schools is an important intervention component. Although these estimates provide some insight into study and program-related effects, they should be taken with caution because they average across multiple sources of between-study heterogeneity. As such, we cannot conclude which characteristics are more important for different mental health, professional burnout, and/or wellbeing outcomes.

Across the breadth and variety of psychological programs included in this review, one consistent similarity emerged. Most programs required significant time, effort, and resources for the teachers to complete. For example, programs were typically delivered face-to-face, in groups in dedicated spaces, and required trained or accredited facilitators who were external to the school. Sessions were also demanding. Most programs included 1–8 sessions that lasted between 1 and 4 h each, delivered over 1–12 weeks, in teachers’ own time. These programs typically followed structured or manualised psychological intervention protocols. For example, mindfulness and/or relaxation-based programs were the most common (39.77%), followed by cognitive-behavioural skills (15.91%) and stress management skills (14.77%). These programs require a significant time commitment for a workforce that is renowned for being time-poor and struggling to cope with ever-changing job demands (Thomson & Hillman, 2020). Time is often identified by teachers as a major barrier to engaging in and completing programs (Fang et al., 2021). Study attrition rates support this as a barrier. Our review showed that attrition rates reached as high as 66.67% in the program groups, with an overall mean of 12.69%. It may be the case the attrition was higher in more demanding programs. We were unable to formally test this hypothesis due to small sample sizes in our meta-analyses, and review findings may be skewed because over 30% of studies did not report attrition.

Programs do not have to be time- or resource-intensive to be effective. For example, providing programs online is one way to reduce demands. This delivery method can be fully automatic, meaning that highly trained experts are not needed to facilitate sessions, and can be asynchronous, meaning that teachers can complete modules in their own time. For example, one study evaluated an online stress management skills program in pre-service, elementary, and secondary school teachers (Ansley et al., 2021). The program was self-paced and consisted of eight 30-min modules, with the recommendation of completing two modules per week for 4 weeks. Most teachers (92.8%) completed the program, defined by the study authors as completing 75% of the modules. The program (versus control) was associated with decreased emotional exhaustion, which is a core feature of professional burnout. Another study evaluated internet-based problem-solving training, consisting of one self-guided lesson per week for 5 weeks, in a teaching sample with elevated depression levels (Ebert et al., 2014). The training (versus control) reduced depressive symptoms, as well as stress and worry, at post-test, 3-month follow-up, and 6-month follow-up. Over half (60%) of teachers completed the program. These completion rates are reasonable given the low intensity of the programs and compare well to rates in other studies evaluating internet-based stress management programs in employee samples (Zarski et al., 2016). Asynchronous online programs may overcome logistical issues in implementation (e.g. organising times, dates, and locations for sessions), prevent premature discontinuation, and be more feasible to roll-out at scale. Despite the potential benefits of such programs, the current review only identified five online programs for teachers. Future research is necessary to develop, evaluate, and establish the effectiveness of online programs to address teacher mental health, professional burnout, and /or wellbeing.

Most of the studies included in the review did not measure program fidelity and program adherence. Fidelity refers to the extent to which a program is delivered as intended (Carroll et al., 2007; Schoenwald et al., 2011) and adherence refers to the extent to which participants completed the program requirements (e.g. all sessions/modules). Fidelity and adherence are important in the translation of research to practice when implementing programs in the real-world. They are key indicators of whether a program is appropriate/acceptable for the intended audience and context, whether there are enough resources to support delivery, and whether it is being used (Carroll et al., 2007). This information is essential to guide effective decisions about which programs to implement, to whom, and when (Peters et al., 2013). Similar to other work in this field (Werner-Seidler et al., 2017), our position is that taking steps to ensure program fidelity and adherence must be a priority in future research. Exploring the extent to which adherence influences outcomes has not yet been investigated in teacher mental health research.

### Methodological Quality

The quality of studies across both RCTs and nRCTs was problematic. Awareness of the assigned intervention, use of self-report measures, absence of a pre-specific analysis plan, and lack of information about missing data and drop out were assessed to be key contributors to bias. Masking intervention assignment in psychological research is difficult because of the inherent nature of psychological interventions; they generally involve a psychoeducational component and require participants to actively learn and engage in new skills. Similarly, using self-report measures in psychological research is the standard approach to assess subjective internal states. Given their ubiquity in the field, awareness and response biases may be less problematic than others identified in our review. Ensuring that methods are transparent and replicable (e.g. by registering protocol and data analysis plans using open science principles) and accurately represent the data (e.g. by using intention to treat analyses and accounting for missing data) will go part way to address bias in analyses and reporting.

Almost half (44.45%) of the studies in the review were nRCTs. While these trials do provide useful information about effectiveness, it is conceptually difficult reach a level of quality that is comparable to an RCT. We classified all nRCTs as having either serious or critical risk of bias. NRCTs are often an initial step in the research process to gauge uptake and response to programs because they are easier to implement that RCTs in real-world contexts. Randomising working teachers, or schools, to different interventions can be difficult, particularly when they are interested in receiving a potentially helpful program. However, our meta-analyses and review showed that effect sizes were generally larger in RCTs than in nRCTs (e.g. *g=*0.63 and *g=*0.33, respectively). RCTs will be an important way forward as the field matures.

### Limitations

The results of this systematic review and meta-analyses need to be interpreted in the context of some limitations. Due to practical reasons, we only included peer-reviewed studies that were published in English and we did not include grey literature or unpublished studies. Another limitation was the small number of studies included in the meta-analyses. There were not enough effect sizes to do more comprehensive meta-analyses or explore moderators. Furthermore, our analyses were likely underpowered to detect small-to-moderate between-group effect sizes for some outcome measures with small sample sizes. Updated meta-analyses are needed once more studies become available in the mental health, professional burnout, and/or wellbeing domains. We also made a series of decisions about which outcome variables to extract and use in the meta-analysis. For example, although we reported on the emotional exhaustion subscale of the Maslach Burnout Inventory, we did not extract other components of burnout (i.e. cynicism, depersonalisation). Although theoretically informed, and guided by our specific aims, these decisions may have introduced a source of researcher bias into the reported results. Finally, given that most available studies reviewed in our paper did not directly examine mechanisms underpinning program effects, we were unable to identify and assess the relative importance of these processes in our analyses.

### Future Research

Our systematic review and meta-analyses identified several areas for future research. Increased consistency in measurement is needed to clarify whether programs influence specific mental health, professional burnout, and/or wellbeing outcomes in the teaching workforce and enable comparison across studies. Increased focus is also needed on less studied, but equally important, outcomes such as trauma, sleep, and resilience. Future research should take a systematic approach to measurement, not only using standardised measures with sound psychometric properties but also those that are validated with teachers and the school context. More comprehensive evaluation of follow-up periods to gauge the trajectory of effects over time once a program has finished is also necessary to further quantify effectiveness. Overall, there is a clear need for rigorous methodology in study design to reduce risk of bias and increase validity and reliability of findings.

Translation from research trials to the real-world is a critical factor to consider when designing and rolling-out programs. Even though the programs identified in our review and meta-analyses are generally effective, their translational potential is unclear. This is particularly the case given how demanding and resource heavy the available programs currently are. Such characteristics are problematic given the high program attrition identified in our review. Insights from co-design research have potential to transform the nature of programs being developed and the studies being designed to evaluate them. Co-design involves relevant stakeholders in the conceptualisation and/or development of programs (Sanders & Stappers, 2008). Most programs identified in our review were developed or adapted for teachers (*n=*67, 76.14%). Although we did not specifically extract and analyse data on co-design, we did not identify any papers that reported co-design principles. Involving teachers, as well as school leadership and representatives from relevant governing bodies, is important to understand what they want and how a program would fit within schools. This process would likely increase the feasibility of implementation, perceived acceptability from those intended to use and benefit from the program and, in turn, the uptake and effectiveness of the program (Steen et al., 2011).

## Conclusions

Our systematic review and meta-analyses identified, described, and evaluated psychological programs targeting the mental health, professional burnout, and/or wellbeing of school teachers. We found that the programs currently available can reduce anxiety, depression, stress and professional burnout, and increase wellbeing, relative to controls. However, there is a need for future studies to prioritise methodological rigour to produce valid results that can be replicated. Most programs currently available require significant time, effort, and resources to deliver and complete. Teachers are time-poor, and these programs may not translate well outside of research trials to real-world contexts. Incorporating principles from co-design and implementation research as well as considering novel ways of delivering programs that reduce demands on teachers (e.g. online programs delivered asynchronously) are necessary to develop programs that work, but that are also used as intended. Against the backdrop of worsening outcomes for teachers due to everchanging roles and responsibilities, as well as unpredictable community events (e.g. COVID-19, other natural disasters), now is the time to develop and evaluate programs that address teacher mental health, professional burnout, and/or wellbeing.

## Supplementary Information

ESM 1 (DOCX 652 KB)

## Footnotes

1 Note that the *n* for the synthesised effect size estimates presented in this section may differ from the total *n* of studies with a certain characteristic because raw data were not available for calculation (see Tables 2 and 4)

2 Based on a series of sensitivity analyses, one study (Ugwoke et al., 2018) was treated as an outlier and removed for burnout. Removing this study almost halved the estimated mean effect size (i.e. from *g=*0*.*98 to *g=*0.57)
