# Supplementary material for: Intervention Programs Targeting the Mental Health, Professional Burnout, and/or Wellbeing of School Teachers: Systematic Review and Meta-Analyses
Source: Educ Psychol Rev. 2023 Mar 1;35(1):26. doi: 10.1007/s10648-023-09720-w (PMC9974401; doi:10.1007/s10648-023-09720-w)
Supplement: Supplementary file 1 — (DOCX 652 KB) [file 10648_2023_9720_MOESM1_ESM.docx]

**Supplementary Material**

Beames et al., 2023, *Educational Psychology Review*

**Appendix A: Search Strategy Example**

PsycINFO:

((((MJMAINSUBJECT.EXACT.EXPLODE("Teachers"))) OR noft(school teacher*) OR noft(teacher*) OR noft(teacher trainee) OR noft(pre-service teacher*) OR noft(educator)) AND (noft(treat*) OR noft(interven*) OR noft(program*) OR noft(train*) OR (noft(("support group" OR "support groups"))) OR noft("professional development")) AND ((MAINSUBJECT.EXACT("Mental Disorders") OR MJMAINSUBJECT.EXACT("Mental Health") OR noft("mental health") OR (noft("mental disorder") OR noft("mental disorders")) OR noft(anxi*) OR noft(mood) OR noft(depress*) OR noft(trauma*) OR noft(sleep) OR noft(insomnia) OR noft(stress) OR noft(distress*) OR noft(burn?out) OR noft(exhaust*) OR noft(well-being) OR noft(wellbeing) OR noft("life satisfaction") OR noft("quality of life"))))

**Appendix B: Figures**

**Fig S1**

*PRISMA Flow Diagram*

**Forest Plots: Randomised Controlled Trials**

**Fig S2**

*Forest Plot for Anxiety*

*
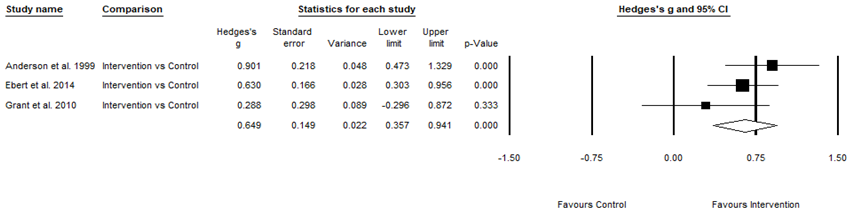
*

**Fig S3**

*Forest Plot for Depression*

*
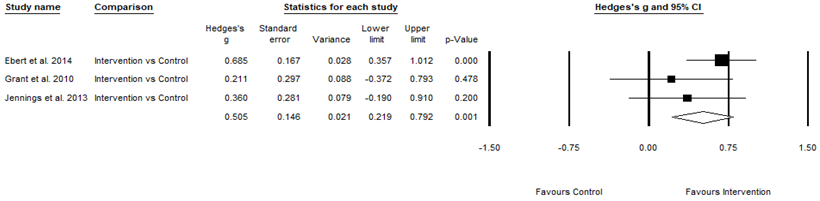
*

**Fig S4**

*Forest Plot for Stress*

*
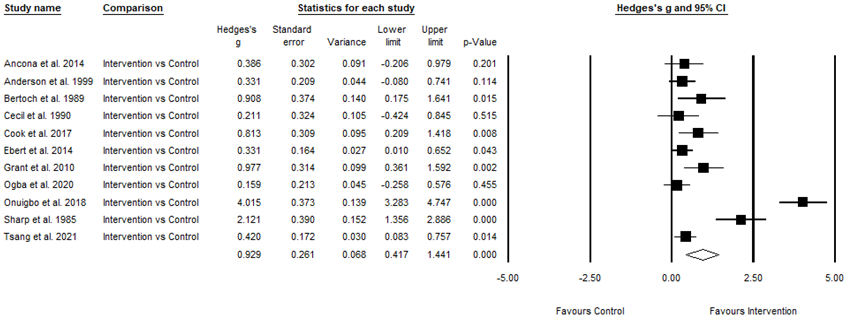
*

**Fig S5**

*Forest Plot for Psychological Distress*

*
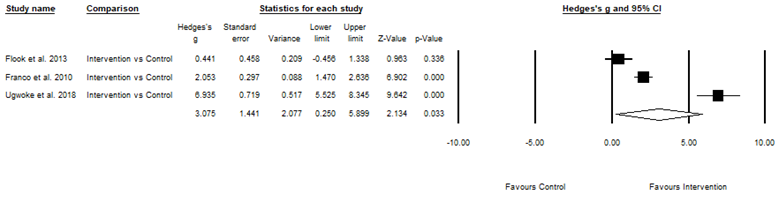
*

**Fig S6**

*Forest Plot for Burnout*

**
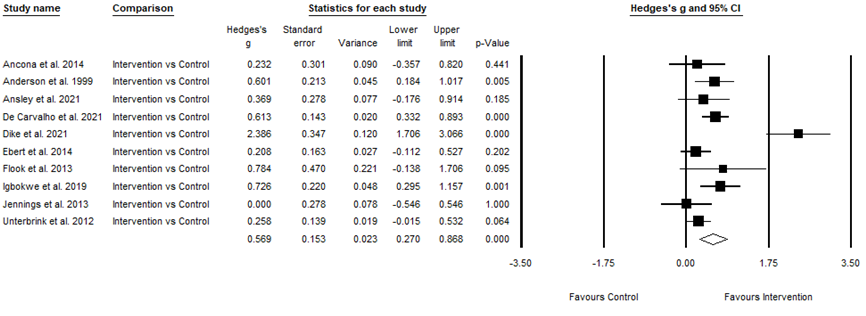
**

**Fig S7**

*Forest Plot for Wellbeing*

 
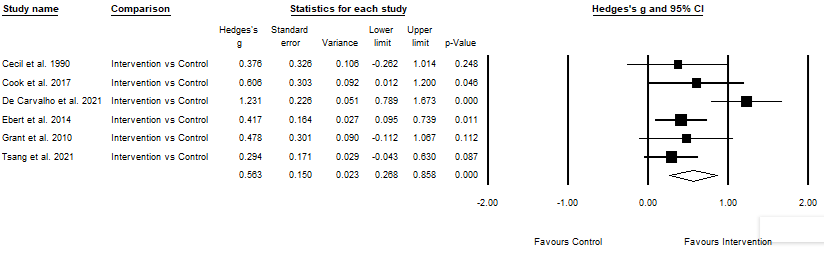


**Forest Plots: Non-Randomised Controlled Trials**

**Fig S8**

*Forest Plot for Anxiety*


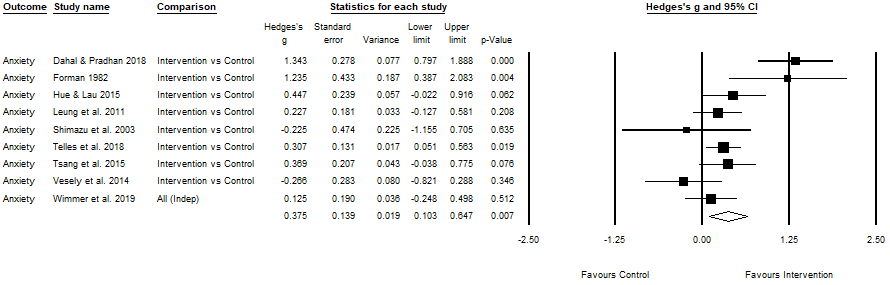


**Fig S9**

*Forest Plot for Depression*


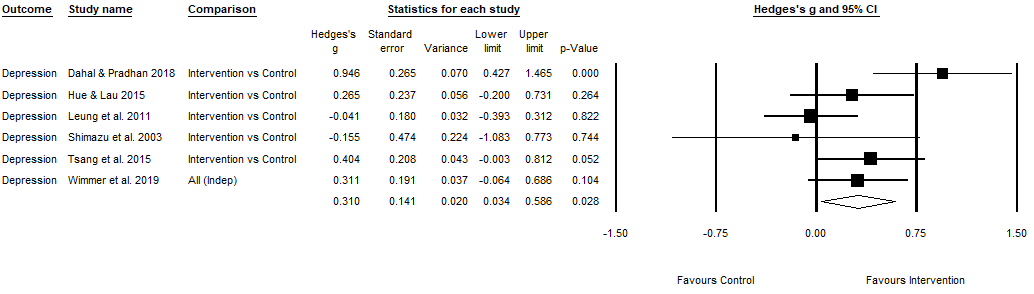


**Fig S10**

*Forest Plot for Stress*


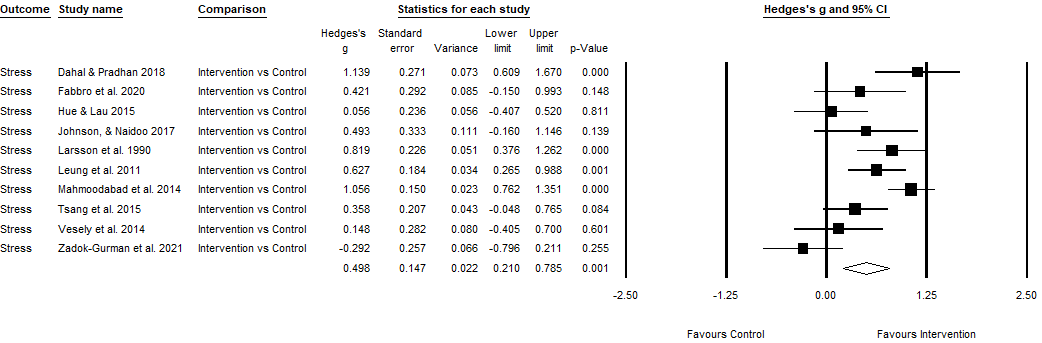


**Fig S11**

*Forest Plot for Burnout*


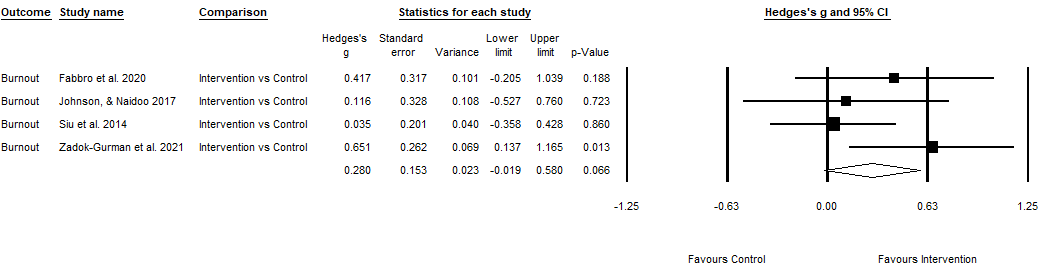


**Fig S12**

*Forest Plot for Wellbeing*


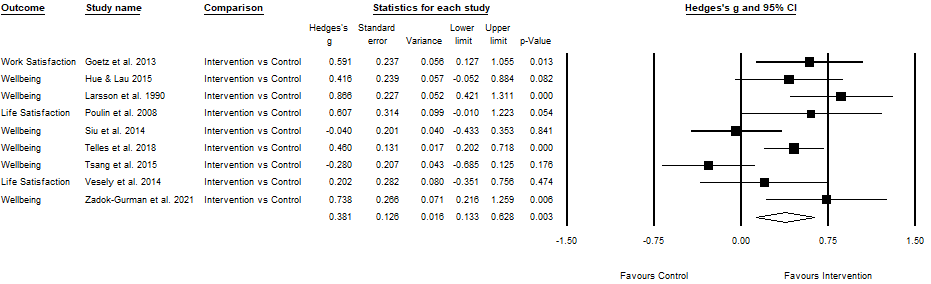


**Fig S13**

*Forest Plot for Somatisation*


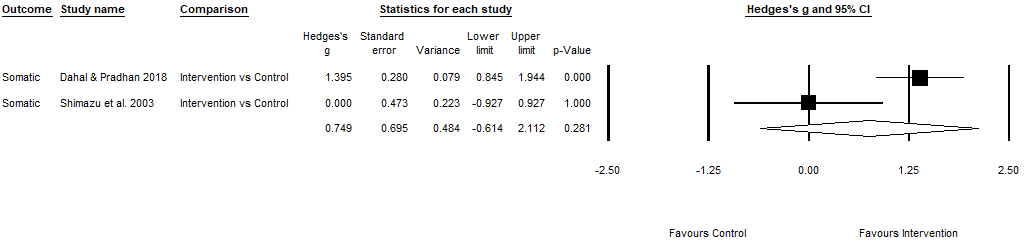


**Appendix C: Tables**

**Table S1**

*Characteristics of Randomised Controlled Trials Included in the Review*

| **Study** | **Country** | **Population** | ***N*** | **Mean Age** | **Attrition** | **Control/Comparison** | **Program** | **Theoretical Approach** | **Program Content** | **Program Sessions** |
| --- | --- | --- | --- | --- | --- | --- | --- | --- | --- | --- |
| Ancona et al. 2014* | USA | Elementary & Middle teachers | 47 | NR | 16.00 | NI | Holistic Life Foundation Yoga & Mindfulness Curriculum | Mindfulness and/or relaxation | Yoga & Mindfulness practices to train awareness and attention, including breathing, visualisation, yoga postures, guided mindful reflection | 3-weeks, six 45-min sessions |
| Anderson et al. 1999* | USA | Elementary, Middle, & Secondary teachers (incl Special Education) | 91 | NR | 0.00 | WL | Standardised Meditation Program | Mindfulness and/or relaxation | Simple mantra meditation, including progressive muscle relaxation, breathing practice, discussion of experiences | 5-weeks, five 1.5-hr sessions delivered weekly plus 1-hr booster session 1-month later |
| Ansley et al. 2021* | USA | Pre-service,  Elementary, & Secondary teachers | 59 | NR | 6.90 | NI | Online Stress Intervention | Stress management skills | Online stress program including response activation and cognitive restructuring, self-care, mindfulness, relaxation, relationships at work, routines and stress, de-escalation | 4-weeks, eight 30-min sessions delivered twice per week |
| Au et al. 2016 | Hong Kong | Elementary Secondary, & Special Education teachers | 60 | NR | 13.33 | Comparison | Stress Management Program | Cognitive-behavioural skills | Cognitive-behavioural principles including psychoeducation, identification of work stress, cognitive restructuring | 8-weeks, eight 90-min sessions delivered weekly |
| Benn et al. 2012^a^ | USA | Special Education teachers & parents | 70 | 45.30 | 34.29 | WL | SMART-in-Education Program | Stress management skills | Stress management and relaxation techniques, based on MBSR plus emotion regulation, forgiveness, kindness, and compassion | 12-weeks, eight 3-hr sessions |
| Berger et al. 2016^a^ | NZ | Secondary teachers, counsellors, administrators, & psychologists | 69 | 44.57 | 2.86 | Comparison | ERASE-Stress New Zealand Program | Other | Coping skills and resilience strategies to deal with traumatic stress, including psychoeducation, mindfulness, body-oriented exercises, art-therapy, narrative techniques | 3 full day workshops |
| Bertoch et al. 1989* | USA | Middle and Secondary teachers | 30 | 38.10 | NR | NI | Stress Management Program | Stress management skills | Psychoeducation, assertiveness training, relaxation and breathing strategies, meditation, self-care | Twelve 2-hr sessions |
| Biglan et al. 2013^a^ | USA | Pre-school teachers, assistants, & affiliated family consultants | 28 | NR | 0.00 | WL | ACT Workshops | ACT | Sleep education, including physiological & behavioural characteristics, mechanisms of regulation, sleep hygiene | 8-weeks, four 4-hr sessions delivered fortnightly |
| Castillo-Gualda et al. 2017^a^ | Spain | Early Childhood, Elementary & Secondary teachers, managers | 54 | 35.98 | 0.00 | NI | RULER Program | Socio-emotional, relationship and/or interpersonal skills | Socio-emotional learning program focused on emotional intelligence skills | 12-weeks, eight 3-hr sessions |
| Cecil et al. 1990^*,b^ | USA | Elementary & Middle teachers | 54 | NR | NR | NI | Stress Inoculation Training | Cognitive-behavioural skills | Cognitive-behavioural principles, including psychoeducation, relaxation training, cognitive restructuring | 6-weeks, six 90-min sessions delivered weekly |
| Chan 2013 | Hong Kong | Teachers (type not specified) | 81 | 33.73 | 2.44 | Comparison | Gratitude Intervention | Other | “Count-your-blessings” activity, involving keeping weekly logs of good things or events | 8-weeks |
| Cheek et al. 2003 | USA | Elementary teachers | 51 | NR | NR | Comparison | Stress Inoculation training | Cognitive-behavioural skills | Cognitive-behavioural principles, including psychoeducation, relaxation training, cognitive restructuring | 6-weeks |
| Cook et al. 2017* | USA | Middle & Secondary teachers | 44 | NR | NR | AC | ACHIEVER Resilience Curriculum | Other | Wellbeing promoting intervention based on positive psychology, cognitive-behavioural principles, and ACT | 5-weeks, five 2.5-hr sessions delivered weekly |
| Cooley et al. 1996^a, b^ | USA | Elementary, Secondary, & Middle Special Education teachers & related services providers | 37 | NR | NR | WL | Stress Management Workshops | Cognitive-behavioural skills | Cognitive-behavioural principles to develop situational, physiological, & cognitive coping skills | 5-weeks, five 2-hr workshops delivered weekly |
| De Carvalho et al. 2021* | Portugal | Elementary teachers | 228 | 43.67 | 8.95 | WL | Mindfulness-Based Program | Mindfulness and/or relaxation | Mindfulness-based program that aims to promote teache’s' social-emotional competencies. Three components: mindfulness practices to reduce stress, emotional self-regulation, and caring practices | 10-weeks, ten 2.5-hour sessions delivered weekly |
| Dike et al. 2021* | Nigeria | Special Education teachers | 58 | 31.95 | 0.00 | WL | Y-CBT Program | Cognitive-behavioural skills | CBT strategies were blended with after-session Yoga exercises. Based on ABCDE group therapeutic modelIncl | 12-weeks, twelve 2-hour sessions delivered weekly |
| Ebert et al. 2014* | Germany | Teachers (type not specified) | 150 | 47.10 | 18.67 | WL | Internet-Based Problem-Solving Training | Cognitive-behavioural skills | Cognitive-behavioural principles, including problem-solving, behavioural activation, coping with rumination | 5 weeks, one self-guided lesson weekly |
| Figl-Hertlein et al. 2014 | Austria | Secondary teachers | 151 | NR | 39.53 | Comparison | Individualised Physiotherapy Directed Occupational Health Program (tailored for teachers) | Stress management skills | Ergonomic and stress management training, including psychoeducation, relaxation strategies, rest and sleep patterns, behavioural activation, body awareness | 5-months, six 30-min sessions |
| Flook et al. 2013* | USA | Elementary teachers | 18 | 43.06 | NR | WL | Modified MBSR (adapted for teachers) | Mindfulness and/or relaxation | Psychoeducation and practices including body scan, sitting meditation, walking meditation, loving-kindness meditation, choiceless awareness, yoga | 8-weeks, eight 2.5-hr sessions + 1 day mindfulness immersion |
| Franco et al. 2010^*^ | Spain | Secondary teachers | 68 | 40.20 | NR | AC | Flow Meditation Program | Mindfulness and/or relaxation | Based on ACT, including learning and practicing flow meditation, allowing and accepting thoughts, sensations and feelings, body-scan, metaphors, mindfulness (breathing) | 10-weeks, ten 90-min sessions |
| Grant et al. 2010* | Australia | Secondary teachers | 44 | 43.21 | NR | WL | Developmental Coaching | Cognitive-behavioural skills | Cognitive-behavioural solution-focused approach to coaching on goal attainment, stress, and workplace wellbeing and resilience | 20-weeks, ten sessions delivered every 1-2 weeks |
| Harris et al. 2016^a^ | USA | Middle teachers & other support staff | 64 | 43.00 | 0.00 | WL | CALM Program for Educators | Mindfulness and/or relaxation | Yoga and mindfulness practices to promote social-emotional competences, stress management, and wellbeing | 16-weeks, sixty-four 20-min sessions delivered 4 days per week |
| Hwang et al. 2019^a^ | Australia | Elementary, Secondary, & Special Education teachers (including non-teaching roles) | 185 | 43.02 | 35.29 | NI | Reconnected | Mindfulness and/or relaxation | MBI, including range of experiential, physical and everyday exercises e.g., mindful yoga, walking, eating and breathing | 8-weeks, eight 90-min sessions |
| Igbokwe et al. 2019* | Nigeria | Pre-service teachers | 96 | 20.72 | 4.17 | NI + Whatsapp group | REBT | REBT | Cognitive, behavioural, and emotive techniques to consciously change irrational thoughts and beliefs, including reframing, distraction, acceptance, rewards | 10-weeks, ten 80-min sessions delivered twice per week |
| Jeffcoat et al. 2012^a^ | USA | Early Education, Elementary, & Secondary teachers, administrators, counsellors, & other staff | 236 | NR | 24.79 | WL | ACT self-help book | ACT | Reading self-help book, *Get out of your mind and into your life,* plus quizzes | 8-weeks, self-guided |
| Jennings et al. 2011 | USA | Pre-service teachers | 43 | 32 | 14.29 | WL | CARE | Mindfulness and/or relaxation | Mindfulness-based professional development program, including emotion skills instruction, mindful awareness, stress reduction practices, caring and listening practices | 6-weeks, four day-long sessions over with intersession phone coaching |
| Jennings et al. 2013* | USA | Early Education, Elementary, Middle, & Secondary teachers | 53 | 36 | 7.41 | WL | CARE | Mindfulness and/or relaxation | Mindfulness-based professional development program, including emotion skills instruction, mindful awareness, stress reduction practices, caring and listening practices | 4-6 weeks, four day-long sessions with intersession phone coaching, plus booster session 2-months later |
| Jennings et al. 2017; 2019 follow up data | USA | Elementary teachers | 224 | NR | 7.00 | WL | CARE | Mindfulness and/or relaxation | Mindfulness-based professional development program, including emotion skills instruction, mindful awareness, stress reduction practices, caring and listening practices | 4-months, five 6-hr training days, plus intersession phone coaching |
| Kaspereen et al. 2012^a^ | USA | Secondary teachers, assistants, receptionists, & coaches | 54 | 40.65 | 0.00 | NI | Structured Relaxation Training customised for teachers | Mindfulness and/or relaxation | Meditation, deep breathing, relaxing music | 4-weeks, four 30-45 min sessions |
| Kemeny et al. 2012 | USA | Teachers (type not specified) | 82 | 41.05 | 7.32 | WL | Contemplative/ Emotion Training | Combination | Secular meditation component including concentration, mindfulness, and directive practices (empathy, compassion); emotional component, including recognising and understanding emotions | 8-weeks, eight all-day/evening sessions (42-hrs total) |
| Mihić et al. 2020^a^ | Croatia | Elementary teachers, counsellors, administrators, & cooks | 54 | 42.55 | 16.70 | WL | CARE for teachers | Mindfulness and/or relaxation | Mindfulness-based professional development program, including emotion skills instruction, mindful awareness, stress reduction practices, caring and listening practices | 3-months, five 6-hr sessions, plus booster session 1-month later |
| Montero-Marin et al. 2021 | England | Secondary teachers | 206 | 18.10 | 38.80 | NI | Instructor-Led Mindfulness Program | Mindfulness and/or relaxation | Introductory skills-based program using mindfulness-based cognitive for improving wellbeing. | 8 weeks, eight 1.5-hour weekly sessions |
| Nwabuko et al. 2019 | Nigeria | Elementary teachers | 86 | NR | NR | NI | REAEI | REBT | Cognitive-behavioural principles, including cognitive restructuring and relaxation, plus relaxation | 16-weeks, 32 2-hr sessions |
| Ogba et al. 2020* | Nigeria | Special Education teachers | 87 | 32.17 | 2.33 | NI | REOHC | REBT | Counselling aimed to counter irrational beliefs, with cognitive, behavioural, and emotive techniques | 12-weeks, 12 1-2 hr sessions delivered weekly |
| Onuigbo et al. 2018* | Nigeria | Elementary Special Education teachers | 86 | 39.38 | 0.00 | NI | REBT | REBT | Cognitive, behavioural, and emotive techniques to dispute irrational beliefs, including cognitive reframing, rational emotive imagery, relaxation, systematic desensitisation | 12-weeks, 24 90-min sessions delivered twice per week |
| Pozo-Rico et al. 2020 | Spain | Elementary teachers | 141 | 38.40 | NR | NI | REBT | REBT | Cognitive, behavioural, and emotive techniques to dispute irrational beliefs, including cognitive reframing, rational emotive imagery, relaxation, systematic desensitisation | 12-weeks, 24 90-min sessions delivered twice per week |
| Rao et al. 2017* | India | Elementary teachers | 60 | 41.50 | 0.00 | NI | MSRT | Mindfulness and/or relaxation | Mindfulness-based, yogic relaxation technique | 4-weeks, 30-min sessions each day, five times per week |
| Roeser et al. 2013 | Canada, USA | Elementary & Secondary teachers | 113 | 46.90 | 10.00 | WL | MT | Mindfulness and/or relaxation | Mindfulness-based, including 5 activities that increase compassion and mindfulness by directing and sustaining attention intentionally and nonjudgmentally on present-moment experiences | 8-weeks, 11 sessions delivered over 36-hours |
| Schloss et al. 1983 | USA | Special Education teachers & associates | 14 | 30 | NR | Comparison | Relaxation Training and Systematic Desensitisation | Stress management skills | Progressive muscle relaxation and gradual exposure to provoking events | 3-months, 32 sessions delivered three times per week |
| Schoeps et al. 2019^a^ | Spain | Early Childhood, Kindergarten, Elementary, & Secondary teachers & counsellors | 340 | 42.64 | NR | NI | Emotional Skills Training | Socio-emotional, relationship and/or interpersonal skills | Emotional abilities and skills to develop perception, expression, understanding, and management of emotions | 3-months, seven 2-hr sessions |
| Sharp et al. 1985*^,b^ | USA | Elementary, Middle, & Secondary teachers | 40 | NR | NR | NI | Stress Inoculation Training adapted for Teachers | Cognitive-behavioural skills | Psychoeducation, skill acquisition including relaxation and rational restructuring, and rehearsal | 4-weeks, eight 2-hr sessions, delivered twice per week |
| Sottimano et al. 2018^b^ | Italy | Pre-school teachers | 318 | 48.30 | 21.59 | NI | Multilevel Approach developed for pre-school teachers | Other | Three approaches including individual psychological counselling, group worksite support intervention (e.g., focusing on group dynamics, relationships with colleagues) and redefinition of work environment, space and furniture | Three 60-min counselling sessions, three 120-min worksite intervention groups, three meetings with architect to redefine workspace |
| Tsang et al. 2021*  (teacher subsample reported here) | Hong Kong | Elementary, & Secondary, Special Education teachers | 186 | 29.55 | 2.15 | WL | MBSR MBCT | Mindfulness and/or relaxation | Based on core elements of MBSR, MBCT and Mindfulness | 8-weeks, eight 1.5-hour weekly sessions. |
| Tunnecliffe et al. 1986^b^ | Australia | Elementary teachers | 14 | NR | NR | WL | Relaxation Training | Mindfulness and/or relaxation | Stress psychoeducation and relaxation training | 5-weeks, five 1.5-2-hr sessions delivered weekly |
| Ugwoke et al. 2018* | Nigeria | Special Education teachers | 54 | 36.67 | 0.00 | WL | RESM | REBT | Behavioural, cognitive, and emotive techniques, including cognitive restructuring, goal-setting, relaxation, motivational interviewing, desensitisation, and imagery | 12-weeks, 20 90-min sessions |
| Unterbrink et al. 2021* | Germany | Secondary teachers | 337 | 46.95 | 34.50 | WL | Professional Relationships Intervention | Socio-emotional, relationship and/or interpersonal skills | Stress psychoeducation, relaxation training, authenticity and role identification, relationships, and social support | 10-months, 10 90-min sessions |
| Wu et al. 2006^*^ | China | Middle teachers | 961 | 50.50 | NR | Control (type NR) | Organisational and Individual Level Stress Management | Stress management skills | Organisational-level strategies to modify/diminish sources of stress at work, including redesigning tasks or flexible working, and individual stress management skills including psychoeducation, relaxation techniques, cognitive coping skills, and work/lifestyle modification skills | Lectures presented twice per month for the first half of the year, then once per month for the remainder |
| Zolnierczyk-Zreda 2005* | Poland | Teachers (type not specified) | 59 | 41.80 | 3.33 | NI | Stress Management Workshop | Stress management skills | Cognitive-behavioural methods of overcoming workload (e.g., time management and priority setting skills), enhancing mastery (e.g., developing positive meaning of work), relationships (e.g., interpersonal and communication skills) | 2-days, 6-hrs per day |

**Note. *** – Studies included in the meta-analysis; **^a^** = includes samples other than teachers, including counsellors/psychologists, administrative staff, principals, parents, affiliated family consultants, managers, learning support etc. *N*, mean ages, and dropout reported in Table 1 for these studies include the whole sample; **^b^** = These studies included more than one intervention group. Only the relevant intervention was selected for inclusion in this review and meta-analysis, which is reported in Table 1 (see “Program” column). The total sample sizes reported for these studies only include the relevant intervention and the control group/s. The mean ages and dropout reported in Table 1 for these studies only include the relevant groups if they were reported. **N** – Total number of participants randomly allocated to groups. **Mean Age** – Mean age of the allocated sample at baseline, unless otherwise specified. **Attrition** – Number of participants lost to drop out in the intervention group only, measured after the final follow up measurement time point. **Control group –** NI=no intervention control, WL=wait-list control, AC=active control. **Program** – ACT=Acceptance and Commitment Therapy; RULER=Recognising, Understanding, Labelling, Expressing, and Regulating Emotion; Y-CBT=Yoga Complemented Cognitive Behavioural Therapy; CBT=Cognitive Behavioural Therapy; MBSR=Mindfulness-Based Stress Reduction; CALM=Community Approach to Learning Mindfully; MBI=Mindfulness-Based Intervention; REBT=Rational Emotive Behaviour Therapy; CARE=Cultivating Awareness and Resilience in Education; REAEI=Rational-Emotive Adult Education Intervention Programme; REOHC=Rational Emotive Occupational Health Coaching; MSRT=Mind Sound Resonance Technique; MT=Mindfulness Training; RESM=Rational-Emotive Stress Management. **NR**=information not reported.

**Table S2**

*Summary of Effect Sizes of Randomised Controlled Trials Included in the Review*

| **Study** | **Outcome Domain/s** | **Outcome Measure/s** | **Effect Size Comparison and Source** | **Effect Size (Hedges’s *g* or Cohen’s *d*)** |
| --- | --- | --- | --- | --- |
| Ancona et al. 2014* | Mental health (stress)  Burnout | TSI  MBI-ES-EE | Program vs NI control at post. Computed from raw data. | *g*=0.39  *g=*0.23 |
| Anderson et al. 1999* | Mental health (stress)  Mental health (anxiety)  Burnout | TSI  STAI-S  MBI-EE | Program vs WL control at post (T2) and 9-week follow-up (T3). Computed from raw data. | T2: *g*=0.33; T3: *g*=0.90  T2: *g*=0.90; T3: *g*=1.40  T2: *g*=0.60; T3: *g*=0.76 |
| Ansley et al. 2021* | Burnout | MBI-ES-EE | Program vs NI control at post. Computed from raw data | *g*=0.37 |
| Au et al. 2016 | Mental health (stress)  Wellbeing | PSS  PWBI-A | Program vs comparison at post (T2) and 4-week follow-up (T3). Computed from raw data. | T2: *g=*0.52; T3: *g*=0.06  T2: *g=*0.14; T3: *g*=-0.23 |
| Benn et al. 2012 | Mental health (anxiety)  Mental health (depression)  Mental health (stress)  Affect (positive)  Affect (negative) | STAI-S  CES-D PSS  PANAS-N  PANAS-P | Program vs WL control at post (T2) and 2-month follow-up (T3). Computed from raw data | T2: *g=*0.44; T3: *g*=0.61  T2: *g=*0.38; T3: *g*=0.49  T2: *g=*0.33; T3: *g*=0.74  T2: *g=*0.39; T3: *g*=0.88  T2: *g=*0.03; T3: *g*=0.37 |
| Berger et al. 2016^a^ | Mental health (post-traumatic stress) Burnout  Resilience | PCL-C  ProQOL-burnout CDRS | Program vs comparison at post (T2) and 8-month follow-up (T3). Computed from raw data. | T2: *g=*0.22; T3: *g*=1.22  T2: *g=*1.25; T3: *g*=0.77  T2: *g=*0.30; T3: *g*=0.43 |
| Bertoch et al. 1989* | Mental health (stress) | OSI | Program vs NI control at post. Computed from raw data. | *g=*0.91 |
| Biglan et al. 2013 | Mental Health (stress) | ITS | Relevant data not available. Cannot compute. | N/A |
| Castillo-Gualda et al. 2017 | Burnout | MBI-EE | Program vs NI control at post. Computed from raw data. | *g=*0.13 |
| Cecil et al. 1990* | Mental health (stress) Wellbeing (job satisfaction) | TSI-EM  JSSS-JS | Program vs NI control at post. Computed from raw data. | T2: *g=*0.21; T3: *g*=-0.12  T2: *g=*0.38; T3: *g*=-0.06 |
| Chan 2013 | Wellbeing (life satisfaction)  Affect (positive)  Affect (negative) | SWLS  PANAS-P  PANAS-N | Program vs comparison at post. Computed from raw data. | *g=*0.40  *g=*0.33  *g=*0.51 |
| Cheek et al. 2003 | Burnout | MBI-ES-EE | Program vs comparison at post. Computed from raw data. | *g=*0.09 |
| Cook et al. 2017* | Mental health (stress)  Wellbeing (work satisfaction) | PSS  SWS | Program vs AC at post. Computed from raw data. | *g=*0.81  *g=*0.61 |
| Cooley et al. 1996 | Burnout  Wellbeing (job satisfaction) | MBI-EE  MSQ-20 | Relevant data not available. Cannot compute. | N/A |
| De Carvalho et al. 2021* | Burnout Wellbeing | MBI-ES-EE MHC-SF | Program vs WL control at post. Computed from raw data. | *g=*0.61  *g=*1.23 |
| Dike et al. 2021* | Burnout | MBI-ES-EE | Program vs WL control at post (T2) and 12-week follow-up (T2). Computed from raw data. | T2: *g*=2.39; T3: *g*=2.19 |
| Ebert et al. 2014* | Mental health (anxiety/worry)  Mental health (depression)  Mental health (stress)  Wellbeing Burnout | PSWQ  CES-D  PSQ  SF-12 MCS  MBI-EE | Program vs WL control at post (T2), 3-month (T3), and 6-month (T4) follow-up. Extracted from text. | T2: *d*=0.63; T3: *d*=0.62; T4: *d=*0.54  T2: *d*=0.59; T3: *d*=0.37; T4: *d=*0.38  T2: *d*=0.36; T3: *d*=0.28; T4: *d=*0.36  T2: *d*=0.40; T3: *d*=0.35; T4: *d=*0.27  T2: *d*=0.24; T3: *d*=0.54; T4: *d=*0.38 |
| Figl-Hertlein et al. 2014 | Wellbeing | SF-36 MCS | Relevant data not available. Cannot compute. | N/A |
| Flook et al. 2013* | Mental health (distress)  Burnout | SC 90-R  MBI-ES- EE | Program vs WL control at post. Computed from raw data. | *g*=0.44  *g*=0.78 |
| Franco et al. 2010* | Mental health (distress) | SC 90-R | Program vs AC at post. Computed from raw data. | *g*=2.05 |
| Grant et al. 2010* | Mental health (anxiety)  Mental health (depression)  Mental health (stress)  Wellbeing  Resilience | DASS-A  DASS-D  DASS-S WWBI  CHS | Program vs WL control at post. Computed from raw data. | *g=*0.29  *g=*0.21  *g=*0.98  *g=*0.48  *g=*0.46 |
| Harris et al. 2016 | Mental health (stress)  Mental health (sleep)  Burnout  Affect (positive)  Affect (negative) | PSS  SRIS  MBI-ES  PANAS-P  PANAS-N | Program vs WL control at post. Computed from raw data. | *g=*0.19  *g=*0.09  *g=*0.08  *g=*0.14  *g=*0.06 |
| Hwang et al. 2019 | Mental health (stress)  Mental health (sleep) | PSS  PSQI | Program vs NI control at post (T2) and 6-week (T3) follow-up. Computed from raw data. | T1: *g=*0.42; T2: *g=*0.45  T1: *g=*0.31; T2: *g=* .29 |
| Igbokwe et al. 2019* | Burnout | OLBI-S-E | Program vs NI + whatsapp group at post (T2) and 3-month (T3) follow-up. Computed from raw data. | T1: *g=*0.73; T2: *g=* 0.73 |
| Jeffcoat et al. 2012 | Mental health (distress) | GHQ-12 | Program vs WL control from baseline to 10-week follow-up. Extracted from text. | *d*=0.73 |
| Jennings et al. 2011 | Mental health (depression)  Affect (positive)  Affect (negative) | CES-D  PANAS-P  PANAS-N | Covariance-adjusted post-program mean comparisons for program vs WL control. Extracted from text. | *d=*0.09  *d=*0.11  *d=*0.43 |
| Jennings et al. 2013* | Mental health (depression)  Burnout  Affect (positive)  Affect (negative) | CES-D MBI-ES-EE PANAS-P  PANAS-N | Program vs WL control at post. Computed from raw data. | *g*=0.36  *g=*0.00  *g*=0.21  *g*=0.23 |
| Jennings et al. 2017 | Mental health (anxiety)  Mental health (depression)  Mental health (stress)  Mental health (sleep)  Burnout  Affect (positive)  Affect (negative) | GAD-7  PHQ-8  PSS  PROMIS-SDQ MBI-ES-EE  PANAS-P  PANAS-N | Relevant data not available. Cannot compute. | N/A |
| Jennings et al. 2019 (follow up data to 2017) | Mental health (anxiety)  Mental health (depression)  Mental health (stress)  Mental health (sleep)  Burnout  Affect (positive)  Affect (negative) | GAD-7  PHQ-8  PSS  PROMIS-SDQ MBI-ES-EE  PANAS-P  PANAS-N | Relevant data not available. Cannot compute. | N/A |
| Kaspereen et al. 2012 | Mental health (stress)  Wellbeing (life satisfaction) | PSS  SWLS | Program vs NI control at post. Computed from raw scores. | *g*=1.20  *g*=0.67 |
| Kemeny et al. 2012 | Mental health (depression)  Affect (positive)  Affect (negative) | BDI  PANAS-P  PANAS-N | Unadjusted mean comparison for program vs WL control at post (T2) and 5-month follow-up (T3). Extracted from text. PANAS effect sizes not reported in text. | T2: *d*=.81; T3: *d*=.90 |
| Mihić et al. 2020 | Burnout | MBI-EE | Program vs WL control at post (T2) and 2-month follow-up (T3). Computed from raw scores. | T2: *g*=0.13; T3: *g*=0.38 |
| Montero-Marin et al. 2021 | Mental health (anxiety)  Mental health (depression)  Mental health (stress) Burnout  Wellbeing | GAD-7  PHQ-9  PSS MBI-ES WEMWBS | Program vs NI control at post. Computed from raw scores. | *g=*0.47  *g=*0.37  *g=*0.58  *g=*0.23  *g=*0.49 |
| Nwabuko et al. 2019 | Burnout | TBI | Relevant data not available. Cannot compute. | N/A |
| Ogba et al. 2020* | Mental health (stress) | OSI | Program vs NI control at post (T2) and 3-month follow-up (T3). Computed from raw data. | T2: *g*=0.16; T3: *g*=0.75 |
| Onuigbo et al. 2018* | Mental health (stress) | TSQ | Program vs NI control at post (T2) and 4-month follow-up (T3). Computed from raw data. | T2: *g*=4.02; T3: *g*=5.35 |
| Pozo-Rico et al. 2020 | Mental health (stress)  Burnout | PSS  MBI | Relevant data not available. Cannot compute. | N/A |
| Rao et al. 2017* | Mental health (anxiety)  Mental health (stress)  Mental health (distress)  Mental health (sleep)  Fatigue | STAI-S  PSS  GHQ-12  PSQI  PFS | Program vs NI control at post. Computed from raw data. | *g=*2.70  *g=*4.94  *g=*1.07  *g=*2.16  *g=*0.76 |
| Roeser et al. 2013 | Mental health (anxiety)  Mental health (depression) Burnout | STAI-S  BDI  MBI | Mean comparison for program vs control at post (T2) and 3-month follow-up (T3). Extracted from text. | T2: *d*=-0.71; T3: *d*=-1.10  T2: *d*=-1.06; T3: *d*=-1.56  T2: *d*=-0.76; T3: *d*=-0.68 |
| Schloss et al. 1983 | Mental health (anxiety) | STAI | Relevant data not available. Cannot compute. | N/A |
| Schoeps et al. 2019 | Mental health (anxiety)  Mental health (depression)  Mental health (stress)  Burnout Wellbeing (life satisfaction) | DASS-A  DASS-D  DASS-S SBI-PE SWLS | Mean comparison for program vs control at post (T2) and 6-month follow-up (T3). Extracted from text. | T2: *d*=-0.09; T3; *d=*-0.22  T2: *d*=-0.17; T3; *d=*-0.04  T2: *d*=-0.06; T3; *d=*-0.17  T2: *d*=-0.43; T3; *d=*-0.75  T2: *d*=-0.52; T3; *d=*-0.21 |
| Sharp et al. 1985* | Mental health (anxiety) | STAI-S | Program vs NI control at post (T2) and 1-month follow-up (T3). Computed from raw data. | T2: *d=*2.12; T3: *d*=2.18 |
| Sottimano et al. 2018 | Burnout | SBI-PE | Relevant data not available. Cannot compute. | N/A |
| Tsang et al. 2021* | Mental health (stress) Wellbeing (life satisfaction)  Affect (positive)  Affect (negative)  Insomnia | PSS SWLS  PANAS-P  PANAS-N  ISI | Program vs WL control at post (T2) and 2-month follow-up (T3). Computed from raw data. | T2: *g*=0.42; T3: *g*=0.57  T2: *g*=0.29; T3: *g*=0.58  T2: *g*=0.50; T3: *g*=0.55  T2: *g*=0.44; T3: *g*=0.67  T2: *g*=0.64; T3: *g*=0.35 |
| Tunnecliffe et al. 1986^b^ | Mental health (stress) | TOSQ | Relevant data not available. Cannot compute. | N/A |
| Ugwoke et al. 2018* | Mental health (distress)  Burnout | PED  TBS-EE | Program vs WL control at post. Computed from raw data. | *g=*6.94  *g=*9.44 |
| Unterbrink et al. 2021* | Burnout | MBI-EE | Program vs WL control at post. Computed from raw data. | *g=*0.26 |
| Wu et al. 2006* | Mental health (stress) | OSI-R-PS | Program vs control at post. Computed from raw data. | *g*=0.04 |
| Zolnierczyk-Zreda 2005* | Burnout | MBI-EE | Program vs NI at post. Computed from raw data. | *g=*0.22 |

**Note. *** – Studies included in the meta-analysis. **Control group –** WL=wait-list control; NI=no intervention control (including treatment as usual); AMC=attention matched control. **Outcome Measures –** MBI-EE=Maslach Burnout Inventory – Emotional Exhaustion Subscale; TSI=Teacher Stress Inventory; PSS=Perceived Stress Scale; PWBI-A=Personal Well Being Index – Adult; STAI-S=State-Trait Anxiety Inventory – State Subscale; CES-D=Center for Epidemiological Studies Depression Scale; PANAS-P=Positive and Negative Affect Scale – Positive; PANAS-N=Positive and Negative Affect – Negative; SBI-PE=Spanish Burnout Inventory – Psychological Exhaustion Subscale; PCL-C=Posttraumatic Check List S, ProQOL=Professional Quality of Life Scale; CDRS=Connor-Davidson Resilience Scale; OSI=Occupational Stress Inventory; JSS=Job Satisfaction Scale; ITS=Index of Teaching Stress; TSI-EM=Teacher Stress Inventory – Emotional Manifestations; JSSS-JS=Job Stress in the School Setting – Job Satisfaction; SWLS=Satisfaction with Life Scale; SWS=Satisfaction with Work Scale; MSQ-20=Minnesota Satisfaction Questionnaire – 20 items; SF-36 MCS=Short Form 36 Health Survey – Mental Health Component Score; SF-12 MCS=Short Form 12 Health Survey – Mental Health Component Score; PSWQ=Penn State Worry Questionnaire; SC 90-R=Symptom Checklist 90-R; MBI-ES=Maslach Burnout Inventory – Educator Survey; MBI-ES-EE=Maslach Burnout Inventory – Educator Survey – Emotional Exhaustion Subscale; DASS-D=Depression Anxiety Stress Scale – Depression Subscale; DASS-A=Depression Anxiety Stress Scale – Anxiety Subscale; DASS-S=Depression Anxiety Stress Scale – Stress Subscale; WWBI=Workplace Well-Being Index; CHS=Cognitive Hardiness Scale; SRIS=Sleep-Related Impairment Scale; PSQI=Pittsburgh Sleep Quality Index; OLBI-S-E=Oldenburg Burnout Inventory for Students – Exhaustion Subscale; PHQ-8=Patient Health Questionnaire 8-item Depression Scale; GAD-7=Generalised Anxiety Disorder 7-item Scale; PROMIS-SDQ=PROMIS Sleep Disorder Questionnaire; WEMWBS=The Warwick-Edinburgh Mental Well-being Scale – 14 items; TBI=Teachers’ Burnout Inventory; OSI=Occupational Stress Index; TSQ=Teacher Stress Questionnaire; GHQ-12=General Health Questionnaire; PFS=Piper Fatigue Scale; BDI=Beck Depression Inventory; SBI-PE=Spanish Burnout Inventory – Psychological Exhaustion Subscale; TOSQ=Teacher Occupational Stress Factor Questionnaire; TBS-E=Teacher Burnout Scale – Exhaustion Subscale; PED=Profile of Emotional Distress; TBS-EE=Teacher Burnout Scale – Emotional Exhaustion; OSI-R-PS=Occupational Stress Index – Revised – Personal Strain Subscale; ISI=Insomnia Severity Index. **Effect Size Comparison and Source –** Hedges’s *g* was computed from raw data extracted from results of primary papers, otherwise Cohen’s *d* was extracted from primary papers when reported. **Effect Size (Hedges’s *g* or Cohen’s *d*) –** Positive effect sizes favour the program over the control.

**Table S3**

*Characteristics of Non-Randomised Controlled Trials Included in the Review*

| **Study** | **Country** | **Population** | ***N*** | **Mean Age** | **Attrition** | **Control/ Comparison** | **Program** | **Theoretical Approach** | **Program Content** | **Program Sessions** |
| --- | --- | --- | --- | --- | --- | --- | --- | --- | --- | --- |
| Berkovich-Ohana et al. 2020 | Israel | Elementary teachers | 39 | NR | 40 | Comparison | MBI | Mindfulness and/or relaxation | Applied mindful pedagogy for educators, including psychoeducation, experiential & mindful practices (e.g., body scan, monitoring experiences) | 3-months, ten sessions delivered over 30-hrs |
| Beshai et al. 2015^a^ | England | Secondary teachers & staff with direct contact with students | 108 | NR | 17.59 | WL | b. Foundations Course | Mindfulness and/or relaxation | Based on MBSR + MBCT | 8-weeks, nine 75-min sessions delivered fortnightly |
| Carroll et al. 2021 | Australia | Elementary & Secondary teachers | 83 | 45.28 | 7.14 | Comparison | MBSR | Mindfulness and/or relaxation | Guided progressive mindfulness meditation practices, body awareness training and gentle yoga | 8-weeks, 2.5-hr sessions delivered weekly (plus a full day retreat in weeks 5 or 6) |
| Castillo-Gualda et al. 2019^a^ | Spain | Kindergarten, Elementary & Secondary teachers & counsellors | 59 | 41.12 | 0.00 | NI | RULER | Socio-emotional, relationship, and/or interpersonal skills | SEL, including emotional awareness, vocabulary, expression, & regulation | 12-weeks, eight 3-hr sessions |
| Cheng et al. 2021 | China | Kindergarten teachers | 70 | 30.96 | 0.00 | NR | Mindfulness Training Program | Mindfulness and/or relaxation | Based on MBSR + MBCT | 4-weeks, four 1.5-hr sessions delivered weekly |
| Cicotto et al. 2014* | Italy | Elementary teachers | 126 | 45.00 | 0.00 | NI | Psychosocial Training | Socio-emotional, relationship, and/or interpersonal skills | Work-related stress & coping strategies, group dynamics, communication, problem-solving, decision-making | 16-weeks, 4 3-hr modules, flexible delivery frequency |
| Dahal & Pradhan 2018* | India | Secondary teachers | 62 | 37.42 | 0.00 | WL | Cyclic Meditation | Mindfulness and/or relaxation | Meditation practice including prayer, relaxation, centering, sitting asanas | 4-weeks, twenty .5-hr sessions delivered daily |
| de Souza et al. 2016* | Brazil | Elementary & Secondary teachers | 84 | 45.50 | 55.17 | WL | Sleep Education Program | Other | Sleep education, including physiological & behavioural characteristics, mechanisms of regulation, sleep hygiene | 8-weeks, four 4-hr sessions delivered fortnightly |
| Dicke et al. 2015*^,b^ | Germany | Pre-service teachers | 61 | NR | 42.86 | WL | Stress Management Training | Stress management skills | Based on German AGIL Training, including Psychoeducation, stress & time management strategies, PMR | Two 8-hr sessions |
| Dyer et al. 2020^a^ | USA | Education professionals (teachers, counsellors, technology specialists, & librarians) | 31 | 47.50 | 22.22 | WL | RISE program | Mindfulness and/or relaxation | Psychoeducation, guided meditation & relaxation, focusing techniques | 3-day residential program, 5-hrs daily |
| Fabbro et al. 2020* | Italy | Kindergarten, Elementary & Middle teachers | 39 | 50.81 | 0.00 | WL | MOM | Mindfulness and/or relaxation | Psychoeducation, mindfulness meditation techniques including mindful breathing, body scan, observation of states of mind | 8-weeks, eight 1.5-hr sessions delivered weekly |
| Figueiredo-Ferraz et al. 2013 | Spain | Elementary teachers | 37 | 46.12 | 0.00 | NI | CBT | Cognitive-behavioural skills | Psychoeducation, cognitive restructuring, physiological and relaxation techniques (e.g., deep breathing, visualisation), emotion regulation techniques | Four 3-4 hr sessions delivered over 4-months |
| Forman 1982* | USA | Secondary teachers | 30 | NR | 33.33 | WL | CB-SMP | Cognitive-behavioural skills | Based on stress inoculation, including psychoeducation, relaxation, cognitive restructuring | 6-weeks, six 3-hr sessions delivered weekly |
| Frank et al. 2015^a^ | USA | Secondary teachers, counsellors, principals, nurses, librarians | 36 | 40.72 | NR | WL | MBSR | Mindfulness and/or relaxation | Mindfulness meditation, including body scan & awareness | 8-weeks, eight 2-hr sessions delivered weekly |
| Friedman et al. 1983 | USA | Elementary & Secondary teachers | 85 | 35.00 | NR | NR | Stress Management | Cognitive-behavioural skills | Cognitive coping skills & relaxation skills for stress | 5-weeks, two to five 2-hr sessions, either delivered weekly or self-directed |
| Goetz et al. 2013* | Germany | Teachers (type not specified) | 95 | 47.40 | 17.64 | NI | AFA Breathing Therapy | Mindfulness and/or relaxation | Holistic approach with focus on perception of breathing, including listening to breath, experiencing the body during movement & relaxation, continuous mindfulness | 11 sessions delivered weekly |
| Gouda et al., 2016 | Germany | Secondary teachers | 29 | 45.90 | 0.00 | WL | MBSR | Mindfulness and/or relaxation | Psychoeducation & mindfulness meditation, including body scan & awareness | 8-weeks, eight 2-hr sessions delivered weekly |
| Hall et al., 1997 | NR | Pre-service teachers | 84^a^ | NR | 2.38^a^ | NI | Human Relations Training | Socio-emotional, relationship, and/or interpersonal skills | Interpersonal skills development, counselling skills, Rogerian style process group | 2-year course (five consecutive 10-week terms), 3-hrs delivered weekly |
| Hue & Lau 2015* | Hong Kong | Pre-service teachers | 135 | 22.32 | 11.56 | NR | MBI | Mindfulness and/or relaxation | Based on MBSR | 6-weeks, six 2.5-hr sessions delivered weekly, plus 1-day retreat (7-hrs) |
| Johnson & Naidoo 2013* | South Africa | Elementary & Secondary teachers | 57 | NR | 10.00 | NI | Transpersonal Psychology Workshops | Other | Emotion-oriented intervention, including mindfulness, visualisation | 6 days, six sessions delivered daily |
| Johnson & Naidoo 2017*^, b^ | South Africa | Secondary teachers | 26 | 46 | 0.00 | NI | Transpersonal Psychology Workshops | Other | Emotion-oriented intervention, including mindfulness, visualisation | 10-weeks, ten 1.5-hr sessions delivered weekly |
|  |  |  |  |  |  |  |  |  |  |  |
| Larsson et al. 1990* | Sweden | Secondary teachers | 118 | 42.33 | 34.12 | NI | Stress Control Programme | Stress management skills | Cognitive-behavioural strategies, including psychoeducation & relaxation exercises | Five to eight 1-3 hr sessions, delivered in 2-3-week intervals |
| Leung et al. 2011* | China | Secondary teachers | 161 | NR | NR | WL | Brief CB-SMP | Cognitive-behavioural skills | Psychoeducation, cognitive-behavioural therapy principles including thought identification and restructuring, pleasurable activities, & relaxation | 1 session |
| Luong et al. 2019* | Germany | Secondary teachers | 96 | 45.16 | 8.00 | WL | MBSR | Mindfulness and/or relaxation | Mindfulness meditation, including body scan & awareness | 8-weeks, eight 2-hr sessions delivered weekly |
| Mahmoodabad et al. 2014* | Iran | Elementary teachers | 200 | 41.65 | NR | NI | Transactional Model Based Teaching Program | Socio-emotional, relationship, and/or interpersonal skills | Cognitive-behavioural strategies, including psychoeducation, coping strategies, self-efficacy, time management, social relationships, physical stress management | Eight 1-1.5 hr sessions |
| Miyahara et al., 2017 | Japan | Pre-service teachers | 60 | NR | 33.33 | Comparison | Sitting Meditation | Mindfulness and/or relaxation | Based on guided mindfulness meditation | One 35-min session |
| Munday et al., 1995 | USA | Pre-service teachers | 35 | NR | NR | NI | Stress Management Training | Stress management skills | Cognitive-behavioural strategies, including psychoeducation, breathing techniques, stretching, cognitive skills, PMR, relaxation, mental quieting | 2-weeks, four sessions (5hr in total), delivered twice per week |
| Poulin et al. 2008* | USA | Pre-service teachers | 44 | 26.35 | 0.00 | NI | MBWE | Mindfulness and/or relaxation | Based on MBSR, with health and wellness promotion component | 8-weeks |
| Schnaider-Levi et al. 2020^*^ | Israel | Secondary teachers | 60 | 46.40 | NR | NI | IBSR | Cognitive-behavioural skills | Cognitive reframing program, including thought identification & challenging, guided reflection | 12-weeks, 12 4.5hr sessions delivered weekly (3.5hr group & 1hr individual) |
| Shimazu et al. 2003* | Japan | Elementary & Secondary teachers | 24 | 44.40 | 33.33 | WL | Stress Management Program | Stress management skills | Combined cognitive behavioural training & relaxation training, including psychoeducation, PMR, coping strategies to manage classroom stressors | Five 2-hr sessions, delivered in 2-4 week intervals |
| Siu et al 2014* | Hong Kong | Elementary & Secondary teachers | 100 | 38.58 | 0.00 | Control (type NR) | Stress Management Program | Stress management skills | Psychoeducation, & skills drawn from cognitive behavioural therapy, emotion regulation, positive psychology, & conflict management (e.g., relaxation, mindfulness, effective communication, & recovery) | 2.5 days, 7-hrs per day |
| Song et al. 2020^a^ | China | Elementary, Middle, Secondary, College, University teachers | 161 | 38.50 | 0.00 | WL | Mindfulness Training | Mindfulness and/or relaxation | Adapted from MBSR, including psychoeducation, sitting meditation, mindful eating, body scan | 4 days, 8hrs per day |
| Telles et al. 2018* | India | Elementary teachers | 236 | 41.90 | 0.00 | NI | Residential Yoga Program | Mindfulness and/or relaxation | Physical postures & exercises, breathing techniques, chanting, guided relaxation | 15 days, 6hrs per day |
| Thephilah et al., 2020 | India | Secondary teachers | 28 | NR | NR | Control (type NR) | Autogenic Relaxation Training | Mindfulness and/or relaxation | Relaxation training, including psychoeducation | 6-weeks, six 2-hr sessions, delivered weekly |
| Todd et al., 2019 | United Kingdom | Elementary & Secondary teachers | 44 | 41.25 | 66.67 | Comparison | MBSR | Mindfulness and/or relaxation | Mindfulness meditation, including body scan & awareness | 8-weeks, eight 2-hr sessions delivered weekly |
| Tsang et al. 2015* | Hong Kong | Elementary teachers | 93 | 38.43 | 21.28 | WL | Stress Management Program | Cognitive-behavioural skills | Based on CBT & CAM, including cognitive restructuring, self-management, relaxation, mindfulness, aromatherapy, acupressure | 6-weeks, six 2-hr sessions |
| Vesely et al. 2014* | Canada | Pre-service teachers | 49 | 26.50 | 0.00 | NI | Managing Occupational Stress through the Development of EI | Socio-emotional, relationship, and/or interpersonal skills | Based on Swinburne EI Model, including emotional self-awareness & expression, reasoning, self-management, management of others, self-control | 5-weeks, five 1.5-hr sessions, delivered weekly |
| Wimmer et al. 2019*^, b^ | Germany | Pre-service teachers | 154 | NR | 7.84 | NI + AMC | MBSR | Combination | Mindfulness meditation, including body scan & awareness, including ACT and Dialectical Behaviour Therapy | 12-weeks, eight 1.5-hr sessions, delivered twice per week |
| Winzelberg & Luskin 1999 | USA | Pre-service teachers | 21 | 24.50 | NR | WL | RISE Program | Mindfulness and/or relaxation | Psychoeducation, guided meditation & relaxation, focusing techniques | 4-weeks, four 45-min sessions, delivered weekly |
| Zadok-Gurman et al. 2021* | Israel | Teachers (type not specified) | 67 | 45.00 | 8.57 | NI | IBSR | Combination | Based on principles and skills of observation, self-exploration, cognitive reframing, and empowerment. | 20-weeks, ten 2.5-hr biweekly group meetings and biweekly 1.5-hr individual sessions |

**Note. *** = Studies included in the meta-analysis; **^a^** = includes samples other than teachers, including counsellors/psychologists, administrative staff, principals, parents, affiliated family consultants, managers, learning support etc. *N*, mean ages, and dropout reported in Table 3 for these studies include the whole sample; **^b^** = These studies included more than one intervention group. Only the relevant intervention was selected for inclusion in this review and meta-analysis, which is reported in Table 3 (see “Program” column). The total sample sizes reported for these studies only include the relevant intervention and the control group/s. The mean ages and dropout reported in Table 3 for these studies only include the relevant groups if they were reported. **N** – Total number of participants allocated to groups. **Mean Age** – Mean age of the allocated sample at baseline, unless otherwise specified. **Attrition** – Number of participants lost to drop out in the intervention group only, measured after the final follow up measurement time point. **Control group –** WL=wait-list control; NI=no intervention control (including treatment as usual); AMC=attention matched control. **Program Content** – MBI=Mindfulness-Based Intervention; MBSR=Mindfulness-Based Stress Reduction; MBWE=Mindfulness-Based Wellness Education; MBCT=Mindfulness-Based Cognitive Therapy; RULER=Recognising, Understanding, Labelling, Expressing, and Regulating Emotion; MOM=Mindfulness-Oriented Meditation; CBT=Cognitive Behavioural Therapy; SEL=Socio-Emotional Learning; PMR=Progressive Muscle Relaxation; CB-SMP=Cognitive Behavioural Stress Management Program; IBSR=Inquiry-Based Stress Reduction; CAM=Complementary and Alternative Medicine; EI=Emotional Intelligence**. NR**=information not reported.

**Table S4**

*Summary of Effect Sizes of Non-Randomised Controlled Trials Included in the Review*

| **Study** | **Outcome Domain/s** | **Outcome Measure/s** | **Effect Size Comparison and Source** | **Effect Size (Hedges’s *g*)** |
| --- | --- | --- | --- | --- |
| Berkovich-Ohana et al. 2020 | Mental health (stress) | PSS | Program vs Comparison at post. Computed from raw data. | *g*=8.07 |
| Beshai et al. 2015 | Mental health (stress) Wellbeing | PSS WEMWBS | Program vs WL control at post. Computed from raw data. | *g=*1.34  *g=*1.34 |
| Carroll et al. 2021 | Mental health (anxiety)  Mental health (depression)  Mental health (stress) Burnout | DASS-A  DASS-D  PSS CBI | Program vs comparison at post (T2) and 5-month follow-up (T3). Computed from raw data. | T2: *g*=0.14; T3: *g*=-0.23  T2: *g*=0.05; T3: *g*=-0.08  T2: *g*=0.00; T3: *g*=0.13  T2: *g*=0.02; T3: *g*=-0.01 |
| Castillo-Gualda et al. 2019 | Burnout | MBI-ES-EE | Program vs NI control at post. Computed from raw data. | *g*=-0.07 |
| Cheng et al. 2021 | Mental health (anxiety)  Mental health (depression)  Mental health (stress) Burnout | DASS-A  DASS-D  DASS-S MBI-EE | Program vs comparison/control (not reported) at post. Computed from raw data. | *g*=0.53  *g*=0.59  *g*=0.37  *g*=0.29 |
| Cicotto et al. 2014* | Wellbeing | BOJSM | Program vs NI control at post. Computed from raw data. | *g*=0.04 |
| Dahal & Pradhan 2018* | Mental health (anxiety)  Mental health (depression)  Mental health (stress) Mental health (somatisation) | GHQ-AI  GHQ-D  PSS  GHQ-SS | Program vs WL control at post. Computed from raw data. | *g*=1.34  *g*=0.95  *g*=1.14  *g*=1.40 |
| de Souza et al. 2016* | Mental health (sleep) | PSQI | Program vs WL control at post. Computed from raw data. | *g*=0.03 |
| Dicke et al. 2015* | Mental health (sleep) Burnout | QSS  MBI-ES-EE | Program vs WL control at post (T2) and 10 to 12-month follow-up (T3). Computed from raw data. | T2: *g*=1.06; T3: *g*=0.26  T2: *g*=0.34; T3: *g*=0.33 |
| Dyer et al. 2020^a^ | Mental health (stress) Burnout Wellbeing (life satisfaction)  Affect (positive)  Affect (negative)  Resilience | PSS  MBI-EE SWLS  PANAS-P  PANAS-N  RS | Program vs WL control at post (T2) and 2-month follow-up (T3). Computed from raw data. | T2: *g*=0.57; T3: *g*=1.26  T2: *g*=-0.29; T3: *g*=0.09  T2: *g*=0.64; T3: *g*=0.77  T2: *g*=-0.87; T3: *g*=0.64  T2: *g*=0.82; T3: *g*=1.17  T2: *g*=0.05; T3: *g*=0.61 |
| Fabbro et al. 2020* | Mental health (stress) Burnout | TSI MBI-ES-EE | Program vs WL control at post. Computed from raw data. | *g*=0.42  *g*=0.42 |
| Figueiredo-Ferraz et al. 2013 | Burnout | SBI-PE | Relevant data not available. Cannot compute. | N/A |
| Forman 1982* | Mental health (anxiety) | STAI-S | Program vs WL control at post. Computed from raw data. | *g*=1.24 |
| Frank et al. 2015 | Mental health (anxiety)  Mental health (depression)  Mental health (somatisation) Mental health (sleep) Burnout | BSI-A  BSI-D  BSI-S  PSQI  MBI-ES-EE | Program vs WL control at post. Computed from raw data. | *g*=0.19  *g*=0.35  *g*=0.29  *g*=2.09  *g*=0.02 |
| Friedman et al. 1983 | Mental health (anxiety)  Mental health (sleep) | STAI-S  SSS | Relevant data not available. Cannot compute. | N/A |
| Goetz et al. 2013* | Wellbeing | AVEM-SW | Program vs NI control at post (T2) and 3-month follow-up. Computed from raw data. | T2: *g*=0.59; T3: *g*=0.00 |
| Gouda et al., 2016 | Mental health (anxiety)  Mental health (depression)  Mental health (stress) | HADS-A  HADS-D  PSQ | Program vs WL control at post. Computed from raw data. | *g*=0.38  *g*=0.29  *g*=-0.61 |
| Hall et al., 1997 | Burnout | MBI-EE | Program vs NI control at post. Computed from raw data. | *g*=0.65 |
| Hue & Lau 2015* | Mental health (anxiety)  Mental health (depression)  Mental health (stress)  Wellbeing | DASS-A  DASS-D  PSS  WHO-5 | Program vs control (type not reported) at post. Computed from raw data. | *g*=0.45  *g*=0.27  *g*=0.06  *g*=0.42 |
| Johnson & Naidoo 2013* | Mental health (anxiety) Burnout | BAI CBI-WS | Program vs NI control at post. Computed from raw data. | *g*=0.56  *g*=0.04 |
| Johnson & Naidoo 2017* | Mental health (stress) Burnout | PSS  CBI-WS | Program vs NI control at post. Computed from raw data. | *g*=0.49  *g*=0.12 |
| Larsson et al. 1990* | Mental health (stress) Wellbeing | SP-ESR SP-GW | Program vs NI control at post. Computed from raw data. | *g*=0.82  *g*=0.87 |
| Leung et al. 2011* | Mental health (anxiety)  Mental health (depression)  Mental health (stress) | DASS-A  DASS-D  DASS-S | Program vs WL control at post. Computed from raw data. | *g*=0.23  *g*=-0.04  *g*=0.63 |
| Luong et al. 2019* | Mental health (anxiety)  Mental health (depression)  Mental health (stress) | HADS-A  HADS-D  PSQ | Program vs WL control at post. Computed from raw data. | *g*=0.28  *g*=-0.07  *g*=0.31 |
| Mahmoodabad et al. 2014* | Mental health (stress) | PSS | Program vs NI control at post (T2) and 1-month follow-up (T3). Computed from raw data. | T2: *g*=1.06; T3: *g*=2.51 |
| Miyahara et al., 2017 | Mental health (stress) | SRS | Program vs comparison at post. Computed from raw data. | *g*=0.48 |
| Munday et al., 1995 | Mental health (anxiety) | STAI-S | Relevant data not available. Cannot compute. | N/A |
| Poulin et al. 2008* | Mental health (psychological distress)  Wellbeing (satisfaction with life) | K10  SWLS | Program vs NI control at post. Computed from raw data. | *g*=0.62  *g*=0.61 |
| Schnaider-Levi et al. 2020* | Mental health (anxiety)  Mental health (depression)  Mental health (stress)  Burnout  Affect (positive)  Affect (negative) | DASS-A  DASS-D  PSS  MBI-EE  PANAS-P  PANAS-N | Program vs NI control at post. Computed from raw data. | *g*=-0.21  *g*=-0.15  *g*=0.40  *g*=0.81  *g*=0.03  *g*=0.15 |
| Shimazu et al. 2003* | Mental health (anxiety)  Mental health (depression)  Mental health (stress)  Fatigue | BJSQ-A  BJSQ-D  BJSQ-SSR  BJSQ-F | Program vs WL control at post. Computed from raw data. | *g*=-0.23  *g*=-0.15  *g*=0.00  *g*=0.40 |
| Siu et al 2014* | Burnout Wellbeing | MBI-EE  ASSET-PW | Program vs control (type not reported) at post. Computed from raw data. | *g*=0.04  *g*=-0.04 |
| Song et al. 2020 | Mental health (stress)  Affect (positive)  Affect (negative) | Chinese PSS  PANAS-P  PANAS-N | Program vs WL control at post. Computed from raw data. | *g*=0.32  *g*=0.15  *g*=0.47 |
| Telles et al. 2018* | Mental health (anxiety) Wellbeing | STAI-S  WEMWBS | Program vs NI control at post. Computed from raw data. | *g*=0.31  *g*=0.46 |
| Thephilah et al., 2020 | Mental health (stress) Burnout  Fatigue | TSI-EM MBI-EE  TSI-FM | Relevant data not available. Cannot compute. | N/A |
| Todd et al., 2019 | Mental health (anxiety)  Mental health (depression)  Mental health (stress) | HADS-A  HADS-D  PSS | Program vs comparison at baseline (T1), post (T2), and 3-month follow-up (T3). | T1: *g*=-0.39; T2: *g=*-0.55; T3: *g=*-0.14  T1: *g*=-0.60; T2: *g=*-0.36; T3: *g=*0.12  T1: *g*=-0.31; T2: *g=*-0.18; T3: *g=*-0.07 |
| Tsang et al. 2015* | Mental health (anxiety)  Mental health (depression)  Mental health (stress)  Wellbeing | DASS-A  DASS-D  DASS-S  OSI-MWS | Program vs WL control at post (T2) and 1-month follow-up (T3). Computed from raw data. | T2: *g=*0.37; T3: *g*=0.24  T2: *g=*0.40; T3: *g*=0.11  T2: *g=*0.36; T3: *g=*0.57  T2: *g*=-0.28; T3: *g=*-0.38 |
| Vesely et al. 2014* | Mental health (anxiety)  Mental health (stress) Wellbeing (life satisfaction)  Resilience | OASIS  PSS SWLS  RSCA-A-R | Program vs NI control at post. Computed from raw data. | *g*=-0.27  *g*=0.15  *g*=0.20  *g*=-0.10 |
| Wimmer et al. 2019* | Mental health (anxiety)  Mental health (depression)  Affect (positive)  Affect (negative) | HADS-A  HADS-D  PANAS-P  PANAS-N | Program vs NI (program vs AMC) at post. Computed from raw data. | *g*=0.19 (*g=*0.06)  *g*=0.38 (*g=*0.25)  *g*=0.25 (*g=*0.10)  *g*=0.25 (*g=*0.00) |
| Winzelberg & Luskin 1999 | Mental health (anxiety)  Mental health (stress) | STAI-S  TSI | Relevant data not available. Cannot compute. | N/A |
| Zadok-Gurman et al. 2021* | Mental health (stress) Burnout Wellbeing  Affect (positive)  Affect (negative)  Resilience | PSS  MBI-EE  PERMA  PANAS-P  PANAS-N  BRS | Program vs NI control at post. Computed from raw data. | *g*=-0.29  *g*=0.65  *g*=0.74  *g*=0.74  *g*=0.12  *g*=0.58 |

**Note. ***=Studies included in the meta-analysis. **Control group –** WL=wait-list control; NI=no intervention control (including treatment as usual); AMC=attention matched control. **Outcome Measures –** PSS=Perceived Stress Scale; WEMWBS=Warwick-Edinburgh Mental Well-Being Scale; MBI-ES-EE=Maslach Burnout Inventory – Educator Survey – Emotional Exhaustion Subscale; MBI-EE=Maslach Burnout Inventory – Emotional Exhaustion Subscale; BOJSM=Brief Overall Job Satisfaction Measure; GHQ-SS=General Health Questionnaire – Somatic Symptoms Subscale; GHQ-AI=General Health Questionnaire – Anxiety and Insomnia Subscale; GHQ-D=General Health Questionnaire – Depression Subscale; PSQI=Pittsburgh Sleep Quality Index; QSS=Quality of Sleep Scale; K10=Kessler 10 Psychological Distress Scale; SWLS=Satisfaction with Life Scale; PANAS-P=Positive and Negative Affect Scale – Positive; PANAS-N=Positive and Negative Affect – Negative; RS=Resilience Scale; TSI=Teacher Stress Inventory; STAI-S=State-Trait Anxiety Inventory – State Subscale; BSI-D=Brief Stress Inventory – Depression Subscale; BSI-A=Brief Stress Inventory – Anxiety Subscale; BSI-S=Brief Stress Inventory – Somatisation Subscale; SSS=Subjective Stress Scale; AVEM-SW=Work-Related Behaviour and Experience Patterns – Satisfaction with Work; PSQ=Perceived Stress Questionnaire; HADS-D=Hospital Anxiety and Depression Scale – Depression Subscale; HADS-A=Hospital Anxiety and Depression Scale – Anxiety Subscale; WHO-5=World Health Organisation-Five Well-Being Index; CBI-WS=Copenhagen Burnout Inventory – Work Subscale; BAI=Beck Anxiety Inventory; SP-ESR=Stress Profile – Emotional Stress Reactions Subscale; SP-GW=Stress Profile – General Wellbeing Subscale; DASS-D=Depression Anxiety Stress Scale – Depression Subscale; DASS-A=Depression Anxiety Stress Scale – Anxiety Subscale; DASS-S=Depression Anxiety Stress Scale – Stress Subscale; SRS=Stress Response Scale; BJSQ-D=Brief Job Stress Questionnaires – Depression Subscale; BJSQ-A=Brief Job Stress Questionnaires – Anxiety Subscale; BJSQ-SSR=Brief Job Stress Questionnaires – Somatic Stress Response Subscale; BJSQ-F=Brief Job Stress Questionnaires – Fatigue Subscale; ASSET-PW=An Organisational Stress Screening Tool – Psychological Well-being Scale; TSI-EM=Teacher Stress Inventory – Emotional Manifestations Subscale; TSI-FM=Teacher Stress Inventory – Fatigue Manifestations; OSI-MWS=Occupational Stress Indicator – Mental Wellbeing Scale; OASIS=Overall Anxiety Severity and Impairment Scale; RSCA-A-R=Resiliency Scale for Children and Adolescence – Adult Version Revised – Emotional Reactivity Subscale; PERMA=Positive Emotion, Engagement, Relationships, Meaning, and Accomplishment scale; BRS=Brief Resilience Scale. **Effect Size Comparison and Source –** Hedges’s *g* was computed from raw data extracted from results of primary papers. **Effect Size (Hedges’s *g*) –** Positive effect sizes favour the program over the control.

**Table S5**

*ROB-II Judgments for each Domain* 

| **Authors** | **Randomisation** | **Deviation** | **Missing** | **Outcome** | **Reporting** | **Overall** |
| --- | --- | --- | --- | --- | --- | --- |
| Ancona et al. 2014 | High | SC | Low | SC | SC | High |
| Anderson et al. 1999 | SC | Low | Low | SC | SC | SC |
| Ansley et al. 2021 | SC | Low | High | SC | SC | High |
| Au et al. 2016 | SC | High | SC | SC | SC | High |
| Benn et al. 2012 | SC | Low | High | SC | SC | High |
| Berger et al. 2016 | Low | SC | Low | SC | SC | SC |
| Bertoch et al. 1989 | SC | SC | SC | SC | SC | SC |
| Biglan et al. 2013 | SC | Low | Low | SC | SC | SC |
| Castillo-Gualda et al. 2017 | SC | Low | Low | SC | SC | SC |
| Cecil et al. 1990 | SC | Low | SC | SC | High | High |
| Chan et al. 2013 | SC | Low | Low | Low | SC | SC |
| Cheek et al. 2003 | SC | Low | High | Low | High | High |
| Cook et al et al. 2017 | SC | Low | High | SC | SC | High |
| Cooley et al. 1996 | SC | Low | High | SC | High | High |
| De Carvalho et al. 2021 | Low | Low | Low | SC | SC | SC |
| Dike et al. 2021 | Low | Low | Low | SC | SC | SC |
| Ebert et al. 2014 | SC | Low | Low | SC | Low | SC |
| Figl-Hertlein et al. 2014 | High | SC | High | SC | SC | High |
| Flook et al. 2013 | SC | Low | High | SC | SC | High |
| Franco et al. 2010 | SC | Low | High | SC | SC | High |
| Grant et al. 2010 | SC | Low | High | SC | SC | High |
| Harris et al. 2016 | High | Low | Low | SC | SC | High |
| Hwang et al. 2019 | High | Low | High | High | SC | High |
| Igbokwe et al. 2019 | SC | SC | Low | SC | SC | SC |
| Jeffcoat et al. 2012 | Low | Low | Low | SC | SC | SC |
| Jennings et al. 2011 | SC | SC | Low | SC | SC | SC |
| Jennings et al. 2013 | SC | SC | Low | SC | SC | SC |
| Jennings et al. 2017 | Low | SC | Low | SC | SC | SC |
| Jennings et al. 2019 | Low | SC | Low | SC | SC | SC |
| Kaspereen et al. 2012 | Low | SC | SC | SC | SC | SC |
| Kemeny et al. 2012 | SC | Low | Low | SC | SC | SC |
| Mihić et al. 2020 | SC | Low | Low | SC | SC | SC |
| Montero-Marin et al. 2021 | SC | Low | Low | SC | SC | SC |
| Nwabuko et al. 2019 | Low | SC | High | SC | SC | High |
| Ogba et al 2020 | Low | Low | Low | SC | SC | SC |
| Onuigbo et al. 2018 | Low | SC | Low | SC | SC | SC |
| Pozo-Rico et al. 2021 | SC | High | High | SC | SC | High |
| Rao et al. 2017 | Low | SC | Low | SC | SC | SC |
| Roeser et al. 2013 | High | SC | Low | High | SC | High |
| Schloss et al. 1983 | SC | SC | High | SC | SC | High |
| Schoeps et al. 2019 | High | SC | High | SC | SC | High |
| Sharp et al. 1985 | High | SC | High | SC | SC | High |
| Sottimano et al. 2018 | SC | Low | Low | SC | SC | SC |
| Tunnecliffe et al. 1986 | High | SC | High | SC | SC | High |
| Tsang et al. 2021 | SC | Low | Low | SC | SC | SC |
| Ugwoke et al. 2018 | Low | Low | Low | SC | Low | SC |
| Unterbrink et al 2012 | Low | High | High | SC | SC | High |
| Wu et al. 2006 | SC | SC | High | SC | SC | High |
| Zolnierczyk-Zreda et al. 2005 | SC | SC | Low | SC | SC | SC |

*Note.* SC=some concerns.

**Table S6**

*ROBINS-I Risk of Bias Judgments for each Domain*

| **Authors** | **Confounding** | **Selection** | **Deviation** | **Missing** | **Outcomes** | **Reporting** | **Overall** |
| --- | --- | --- | --- | --- | --- | --- | --- |
| Berkovich-Ohana et al. 2020 | Serious | Low | Low | NI | Serious | Serious | Serious |
| Beshai et al. 2015 | Moderate | Low | Low | Serious | Serious | Low | Serious |
| Carroll et al. 2021 | Moderate | Low | Low | Low | Serious | Low | Serious |
| Castillo-Gualda et al. 2019 | Moderate | Low | Low | Low | Serious | Serious | Serious |
| Cicotto et al. 2014 | Serious | Low | Low | Low | Serious | Serious | Serious |
| Cheng et al. 2021 | Serious | Low | Low | Low | Serious | Low | Serious |
| Dahal & Pradhan 2018 | Moderate | Low | Low | NI | Serious | Low | Serious |
| de Souza et al. 2016 | Serious | Low | Low | Serious | Serious | Serious | Serious |
| Dicke et al. 2015 | Moderate | Low | Low | Low | Serious | Low | Serious |
| Dyer et al. 2020 | Serious | Low | Low | Serious | Low | Serious | Serious |
| Fabbro et al. 2020 | Serious | Low | Low | Low | Serious | Low | Serious |
| Figueiredo-Ferraz et al. 2013 | Critical | Low | Low | Low | Serious | Low | Critical |
| Forman 1982 | Serious | Low | Low | Serious | Serious | Moderate | Serious |
| Frank et al., 2015 | Moderate | Low | Low | NI | Serious | Low | Serious |
| Friedman et al., 1983 | Moderate | Low | Low | NI | Serious | Serious | Serious |
| Goetz et al. 2013 | Moderate | Low | Low | Serious | Serious | Low | Serious |
| Gouda et al., 2016 | Moderate | Low | Low | Low | Serious | Low | Serious |
| Hall et al., 1997 | Serious | Low | Low | Low | Serious | Serious | Serious |
| Hue & Lau 2015 | Moderate | Low | Low | Serious | Serious | Low | Serious |
| Johnson & Naidoo 2017 | Moderate | Low | Low | Low | Serious | Low | Serious |
| Johnson & Naidoo 2013 | Moderate | Low | Low | Low | Serious | Serious | Serious |
| Larsson et al. 1990 | Serious | Low | Low | Serious | Serious | Serious | Serious |
| Leung et al. 2011 | Moderate | Low | Low | Critical | Serious | Serious | Critical |
| Luong et al. 2019 | Moderate | Low | Low | Low | Serious | Low | Serious |
| Mahmoodabad et al. 2014 | Moderate | Low | Low | NI | Serious | Serious | Serious |
| Miyahara et al., 2017 | Serious | Low | Low | Low | Serious | Low | Serious |
| Munday et al., 1995 | Serious | Low | Low | NI | Serious | Serious | Serious |
| Poulin et al. 2008 | Critical | Low | Low | Low | Serious | Low | Critical |
| Schnaider-Levi et al. 2020 | Moderate | Low | Low | NI | Serious | Low | Serious |
| Shimazu et al. 2003 | Serious | Low | Low | Serious | Serious | Low | Serious |
| Siu et al 2014 | Critical | Low | Low | Low | Serious | Serious | Critical |
| Song et al. 2020 | Serious | Low | Low | NI | Serious | Serious | Serious |
| Telles et al. 2018 | Serious | Low | Low | Low | Serious | Serious | Serious |
| Thephilah et al., 2020 | Serious | Low | Low | NI | Serious | Critical | Critical |
| Todd et al., 2019 | Serious | Low | Low | Serious | Serious | Low | Serious |
| Tsang et al. 2015 | Moderate | Low | Low | Low | Serious | Low | Serious |
| Vesely et al. 2014 | Serious | Low | Low | Low | Serious | Serious | Serious |
| Wimmer et al. 2019 | Moderate | Low | Low | Serious | Serious | Low | Serious |
| Winzelberg & Luskin 1999 | Serious | Low | Low | Moderate | Serious | Serious | Serious |
| Zadok-Gurman et al. 2021 | Moderate | Low | Low | Moderate | Serious | Low | Serious |

*Note.* NI=no information.

**Table S7**
*RCT Meta-Analytic Results at Post-Intervention and Short-Term Follow-Up*

| **Primary Outcomes** | *Hedges’s g* | *k* | *p* | CI_lower_ | CI_upper_ |
| --- | --- | --- | --- | --- | --- |
| *Post-Intervention* |  |  |  |  |  |
| Depression | 0.51 | 3 | .001* | 0.22 | 0.79 |
| Anxiety | 0.65 | 3 | <.001* | 0.36 | 0.94 |
| Stress | 0.93 | 11 | .001* | 0.42 | 1.44 |
| Psychological distress | 3.08 | 3 | .033 | 0.25 | 5.90 |
| Burnout | 0.57 | 10 | <.001* | 0.27 | 0.87 |
| Wellbeing | 0.56 | 6 | <.001* | 0.27 | 0.86 |
| *Short Term Follow-Up* |  |  |  |  |  |
| Anxiety | 1.48 | 3 | 0.01 | 0.31 | 2.65 |
| Stress | 1.79 | 8 | .001* | 0.87 | 2.71 |
| Sleep | 1.24 | 2 | .17 | -0.53 | 3.01 |
| Burnout | 0.82 | 5 | .002* | 0.30 | 1.33 |
| Wellbeing | 0.33 | 3 | .04 | 0.02 | 0.64 |
| **Secondary Outcomes** |  |  |  |  |  |
| *Post-Intervention* |  |  |  |  |  |
| Positive Affect | 0.42 | 2 | .004* | 0.13 | 0.71 |
| Negative Affect | 0.38 | 2 | .009 | 0.01 | 0.67 |

*Note.* *Significant at the conservative threshold of *p*<.008.

**Table S8**
*RCT Heterogeneity Statistics for the Primary Analyses at Post-Intervention*

|  | **Heterogeneity Statistics** | | | | | |
| --- | --- | --- | --- | --- | --- | --- |
|  | *Q* | *df* | *p* | *I*^2^ | CI_lower_ | CI_upper_ |
| **Primary Outcomes** |  |  |  |  |  |  |
| Depression | 2.38 | 2 | .31 | 15.81 | 0.00 | 97.18 |
| Anxiety | 2.81 | 2 | .25 | 28.83 | 0.00 | 97.61 |
| Stress | 113.01 | 10 | <.001 | 91.15 | 86.19 | 94.33 |
| Psychological distress | 58.31 | 2 | <.001 | 96.57 | 92.88 | 98.35 |
| Burnout | 42.16 | 9 | <.001 | 78.65 | 61.17 | 88.26 |
| Wellbeing | 12.34 | 5 | .03 | 59.49 | 0.36 | 83.52 |
| **Secondary Outcomes** |  |  |  |  |  |  |
| Positive Affect | 0.82 | 1 | .37 | 0.00 | - | - |
| Negative Affect | 0.41 | 1 | .52 | 0.00 | - | - |

*Note.* Confidence interval estimates cannot be calculated when *df*<2.

**Table S9**
*nRCT Meta-Analytic Results at Post-Intervention and Short-Term Follow-Up*

| **Primary Outcomes** | *Hedges’s g* | *k* | *p* | CI_lower_ | CI_upper_ |
| --- | --- | --- | --- | --- | --- |
| *Post-Intervention* |  |  |  |  |  |
| Depression | 0.31 | 6 | .03 | 0.03 | 0.59 |
| Anxiety | 0.38 | 9 | 0.007* | 0.10 | 0.65 |
| Stress | 0.50 | 10 | <.001* | 0.21 | 0.79 |
| Burnout | 0.28 | 4 | .07 | -0.19 | 0.58 |
| Wellbeing | 0.38 | 9 | .003* | 0.13 | 0.63 |
| Somatisation | 0.75 | 2 | .28 | -0.61 | 2.11 |
| *Short Term Follow-Up* |  |  |  |  |  |
| Depression | 0.02 | 2 | .88 | -0.27 | 0.31 |
| Anxiety | 0.33 | 3 | .01 | 0.08 | 0.58 |
| Stress | 1.14 | 3 | .12 | -0.28 | 2.54 |
| Burnout | 0.14 | 2 | .51 | -0.28 | 0.57 |
| Wellbeing | -0.11 | 3 | .40 | -0.38 | 0.15 |
| **Secondary Outcomes** |  |  |  |  |  |
| *Post-Intervention* |  |  |  |  |  |
| Positive Affect | 0.43 | 2 | .13 | -0.13 | 0.98 |
| Negative Affect | 0.12 | 2 | .44 | -0.18 | 0.42 |
| Resilience | 0.25 | 2 | .46 | -0.42 | 0.92 |

*Note.* *Significant at the conservative threshold of *p*<.008.

**Table S10**
*nRCT Heterogeneity Statistics for the Primary Analyses at Post-Intervention*

|  | **Heterogeneity Statistics** | | | | | |
| --- | --- | --- | --- | --- | --- | --- |
|  | *Q* | *df* | *p* | *I*^2^ | CI_lower_ | CI_upper_ |
| **Primary Outcomes** | | | | | | |
| Depression | 10.71 | 5 | .06 | 53.33 | 0.00 | 81.34 |
| Anxiety | 25.23 | 8 | .001 | 68.29 | 32.35 | 84.20 |
| Stress | 35.77 | 9 | <.001 | 74.84 | 53.05 | 86.52 |
| Burnout | 3.92 | 3 | .27 | 23.40 | 0.00 | 87.93 |
| Wellbeing | 22.06 | 8 | .003 | 65.32 | 29.36 | 82.96 |
| Somatisation | 6.44 | 1 | .01 | 84.47 | - | - |
| **Secondary Outcomes** |  |  |  |  |  |  |
| Positive Affect | 3.04 | 1 | .08 | 67.09 | - | - |
| Negative Affect | 0.00 | 1 | .99 | 0.00 | - | - |
| Resilience | 3.12 | 1 | .08 | 67.97 | - | - |

*Note.* Confidence interval estimates cannot be calculated when *df*<2.
